# Molecular Inversion Probes for targeted resequencing in non-model organisms

**DOI:** 10.1038/srep24051

**Published:** 2016-04-05

**Authors:** M. Niedzicka, A. Fijarczyk, K. Dudek, M. Stuglik, W. Babik

**Affiliations:** 1 Institute of Environmental Sciences, Jagiellonian University, Gronostajowa 7, 30-387 Kraków, Poland

## Abstract

Applications that require resequencing of hundreds or thousands of predefined genomic regions in numerous samples are common in studies of non-model organisms. However few approaches at the scale intermediate between multiplex PCR and sequence capture methods are available. Here we explored the utility of Molecular Inversion Probes (MIPs) for the medium-scale targeted resequencing in a non-model system. Markers targeting 112 bp of exonic sequence were designed from transcriptome of *Lissotriton* newts. We assessed performance of 248 MIP markers in a sample of 85 individuals. Among the 234 (94.4%) successfully amplified markers 80% had median coverage within one order of magnitude, indicating relatively uniform performance; coverage uniformity across individuals was also high. In the analysis of polymorphism and segregation within family, 77% of 248 tested MIPs were confirmed as single copy Mendelian markers. Genotyping concordance assessed using replicate samples exceeded 99%. MIP markers for targeted resequencing have a number of advantages: high specificity, high multiplexing level, low sample requirement, straightforward laboratory protocol, no need for preparation of genomic libraries and no ascertainment bias. We conclude that MIP markers provide an effective solution for resequencing targets of tens or hundreds of kb in any organism and in a large number of samples.

High-throughput sequencing has become an indispensable research tool in ecology and evolutionary biology^1^,^2^. Whole genome de novo sequencing, assembly and resequencing at a population scale are currently feasible for many non-model species^3^. However whole genome resequencing (WGR) is a costly and challenging endeavor in no small part due to data storage, curation and analysis issues. Also, WGR still remains beyond reach for organisms with particularly large or complex genomes, such as many insects, amphibians or plants. More importantly, WGR is not necessary for addressing many questions which still do require information about genomic patterns of variation. Examples include species delimitation, phylogeographic inferences, assessment of genetic structure and gene flow, estimation of genetic variation within populations as well as studies focusing on predefined gene sets or involving construction of linkage maps. For such applications it is sufficient to sample many loci that collectively represent a fraction of the genome, at a fraction of the WGR cost. Hence there has been a wide interest in reduced representation (genome-partitioning) techniques which provide such markers^4^,^5^.

Reduced representation approaches may be broadly divided into two classes which complement each other and have been used effectively to address consequential evolutionary and ecological questions^6^,^7^,^8^. The first class comprises techniques that sample the genome approximately at random; the researcher may control the size but not identity of the target. Various genotyping by sequencing approaches relying on restriction enzymes, for example restriction site associated DNA sequencing (RADseq), fall into this class^9^,^10^. The second class encompasses a diverse array of methods which give the researcher some control over the identity or the functional class of the assayed portion of the genome, be it transcribed sequences (RNAseq), transcription factor binding sites (ChipSeq) or transcriptionally active chromatin (DNaseSeq). The highest degree of control over the identity of the interrogated regions is provided by targeted resequencing methods^11^. Sequence capture approaches which rely on hybridization of genomic DNA to numerous probes of known sequence, have proved especially popular^12^,^13^,^14^.

Hybridization-based targeted resequencing methods, although immensely powerful, have limitations which make them less than ideal choice in some situations. These methods are technically demanding, require construction of genomic libraries prior to hybridization, are time consuming and do not scale well with the number of samples. They are not very efficient when the target is small, on the order of tens of kilobases (kb), or when genome size is very large, because typically obtained enrichment rates are hundreds-fold^15^. Yet applications in which tens, hundreds or thousands of defined genomic regions need to be interrogated in a large number of samples are common. They include construction of linkage maps or incorporation of new genes into the existing maps^16^, studies of natural hybridization and introgression in hybrid zones^17^ and genotyping sets of candidate genes in ecological genomics studies^18^,^19^. Such applications fall in between PCR-based methods characterized by high specificity but low multiplexing capabilities and sequence capture methods with their megabase-size targets. A considerable interest in such intermediate scale of targeted resequencing in biomedicine has led to the development of various commercial solutions which are however not available in non-model organisms^20^.

Molecular Inversion Probes (MIP)^21^ appear particularly well suited for targeted resequencing of tens, hundreds or thousands of short genomic regions. They can be used in any organism with partial genomic information available. MIPs are single-stranded DNA molecules containing on their ends sequences complementary to two regions flanking the target of up to several hundred bp. Following hybridization of MIPs to the target, gap-filling and ligation result in circularized DNA molecules containing sequence of the target together with adaptors and barcodes ready for downstream analyses (Fig. 1). MIP technique was a popular solution at early stages of large-scale human SNP genotyping^22^. More recently MIPs have been used for resequencing large sets of human exons^23^ and medically relevant gene panels^24^. Specialized applications of MIPs include detection of low frequency variants^25^, copy-number variation (CNV)^24^,^26^, accurate genotyping of highly similar paralogs^27^ and quantification of alternative splicing^28^.

So far MIP markers have not been widely used in research on non-model organisms. Yet they offer a number of potential advantages for ecological and evolutionary research. Therefore we explored their utility in a non-model system by assessing performance of MIP markers designed from transcriptome sequences of *Lissotriton* newts.

## Results

### MIP performance and rebalancing

The workflow for MIP design and analysis is summarized in Fig. 2. The markers were designed to include positions identified through transcriptome resequencing as diagnostic for *Lissotriton montandoni* and *L. vulgaris* (see Methods) and thus useful for constructing a linkage map. In the experiment 1 we tested performance of 248 MIPs in 24 individuals under equal concentration of all probes. We obtained 15.4 mln paired-end Illumina reads, on average 77.3% (SD 4.7%) reads were on target and the mean coverage was 2080×. No reads were obtained for 14 MIPs (5.6%) and these were excluded from further analyses. Performance of individual MIPs was expressed as the Fraction of Mapped Reads (FMR) within sample. Ideally, if capture efficiency were uniform, all MIPs should have similar FMR both within and among individuals. The distribution of median FMR as well as variation among individuals for the 234 MIPs with reads on target are shown in Fig. 3. The medians of 187 MIPs (80%) were within one order of magnitude (FMR 0.0010–0.0094). Performance of individual MIPs across samples was more uniform: for 218 MIPs (93%) the difference between the 10^th^ and 90^th^ FMR percentile was less than 5-fold; such uniformity was obtained for 97% of 187 MIPs mentioned above. Targets of 65 MIPs were longer than the standard 112 bp. No significant correlation between the median FMR and target length was detected (Spearman’s *ρ* = −0.093, *P* = 0.16).

**Figure 1:**
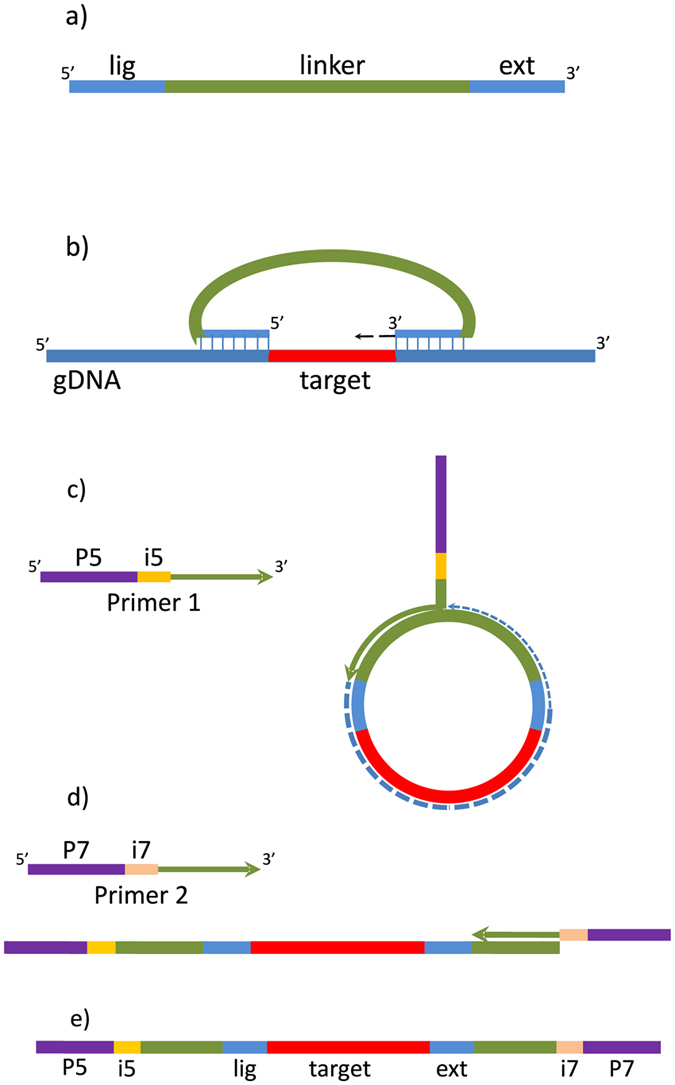
Molecular Inversion Probe (MIP) and the principle of the method; (**a**) the structure of a MIP; lig -ligation arm, ext -extension arm, (**b**) hybridization of the MIP to the target, gap-filling and ligation, (**c**) 1st cycle of library amplification, P5–P5 Illumina adaptor, i5 - Nextera index, (**d**) 2nd cycle of library amplification, P7–P7 Illumina adaptor, i7 - Nextera index, fragments of the Primers 1 and 2 complementary to the custom sequencing primers (Table S1) are in green, (**e**) the final product ready for Illumina sequencing.

The experiment 2 was performed using the rebalanced MIP pool, with probe-to-target ratio increased for the 24 worst performing (median FMR <0.001) markers, and decreased for the 1 best performing (FMR* *= 0.04) MIP; 23.3 mln reads were obtained. Rebalancing indeed improved performance of rebalanced MIPs (Mann-Whitney paired test, *V *= 23, *P *= 1.5 × 10^−4^; Fig. 4a). However rebalancing did not significantly reduce FMR variance among MIPs (Fig. 4b, Levene’s test on medians, *P *= 0.84). A surprising consequence of rebalancing was reduction of specificity as only 37.3% (SD 12.7%) reads were on target; the mean coverage in the experiment 2 was 516×. The comparison of gel pictures of amplified pools before and after rebalancing clearly shows increase of nonspecific amplification products (Fig. S1). Even though target bands were excised from gel and purified, Bioanalyzer traces (Fig. S2) show that the peak centered on the expected length of 270 bp (target + arms + Illumina adaptors, Fig. 1) was broader than before rebalancing, which may indicate increased fraction of nonspecific products of the length similar to the target.

### Genotyping and validation of MIPs as single locus Mendelian markers

Among 234 MIPs with reads on target, genotypes could not be called for 3 markers due to low coverage and excessive number of mismatches. There was a significant negative correlation between the coverage per individual and the number of missing genotypes (experiment 1: Spearman’s *ρ *= −0.504, *P *= 0.012, experiment 2: *ρ *= −0.719, *P *= 1.7 × 10^−13^). Tests of Mendelian inheritance were performed for 216 out of 231 genotyped MIPs (93.5%) with no missing data and containing at least one polymorphic site within the family (parents + 21 offspring, 1622 sites in total). Markers in which at least one polymorphic site had *P *< 0.015 (one of genotypes expected according to the Mendelian segregation rules completely missing) were marked as potential paralogs. This procedure flagged 25 MIP markers (11.6% of all tested) as potential paralogs. Thus 191 (77% of the initial 248) markers were confirmed as polymorphic and single copy.

Tests of the excess and deficit of heterozygotes were performed in three natural *Lissotriton vulgaris graecus* (*Lvg*) populations to further check for the presence of potential paralogs and identify loci with null alleles. 164 MIPs were polymorphic in *Lvg* (in total 601 polymorphic sites). We detected 91 sites in 20 MIPs (12.2% of all tested markers) showing an excess of heterozygotes at the false discovery rate (FDR) 0.05. These markers were also flagged as potential paralogs and removed from further analyses; 12 (36% of all) potential paralogs were marked as such in both family-based and population-based analyzes. Deficit of heterozygotes suggesting the presence of null alleles was detected at FDR 0.05 for 59 sites in 8 MIPs (4.8%). The non-reference discrepancy rate (NRD) estimated for 16 *Lvg* individuals genotyped in replicates was 0.008 (SD 0.0076) indicating >99% genotyping concordance.

The three *Lvg* populations differed greatly in the level of genetic variation (Table 1). Both the number of segregating sites and nucleotide diversity were the lowest in the Milia population in the Peloponnese (*S *= 36, π* *= 0.0003), and the highest in the Gracen population in Albania (*S *= 224, π* *= 0.0024).

## Discussion

In this study we tested performance of Molecular Inversion Probes (MIP) as molecular markers in non-model species without a sequenced genome. The markers were designed from transcriptome sequences but were genotyped from genomic DNA. Hence identification of exon boundaries in transcripts was essential for successful genotyping^29^,^30^. We applied a homology-based approach which relies on the observation that most exons, especially constitutive ones, are conserved across vertebrates^31^,^32^. Indeed the exon boundaries were correctly identified in most newt protein-coding transcripts using gene models of *Xenopus*, which diverged from newts ca. 300 mya^33^. The ca. 5% MIPs without mapped reads are the likely cases of inaccurate prediction of exon boundaries. These failures could be due to incorrect identification of orthologs, the lack of conservation of exon-intron boundaries between *Xenopus* and newts or because of erroneous identification of exon boundaries by our blastn-based scripts.

Considering only markers with mapped reads, important measures of their performance are specificity and coverage uniformity. We targeted ca. 28 kb of genomic sequence which is less than 10^−6^ of the 30 Gb *Lissotriton* genome^34^. With 77% reads on target the enrichment rate was almost million-fold, approaching that of PCR. Specificity was lower than 98–99% reported in humans^11^,^23^, but when the fraction of reads mapped to reference is taken into account, the difference between results obtained for humans (89.5%) and newts (77.3%) is not large. Coverage uniformity is lower for MIP markers than for other targeted resequencing methods, a major limitation of the technique^11^. In our study uniformity (80% of MIPs within the 10-fold range) compares favorably with that reported in a human study utilizing a large set of array-synthesized MIPs (58% of MIPs within the 10-fold range)^23^ and is similar to that for column-synthesized MIPs after rebalancing (90% of MIPs within the 15-fold range)^24^. In our hands rebalancing by increasing concentration of poor performers 100-fold^25^ slightly improved performance of the rebalanced probes but decreased the fraction of reads on target; such effect has not been reported in human studies. It is possible that increasing concentration of poor performers 10 or 50 fold^24^ would produce better results. However it is worth noting that even without rebalancing uniformity was acceptable. Therefore, although further tests of rebalancing may be desirable, we suppose that in many situations discarding poorly performing markers may be a satisfactory solution. In accordance with earlier reports^23^ capture efficiencies of individual MIPs were highly reproducible. Thus if samples are sequenced to similar coverage, the fraction of missing data should be low for most MIPs. This is an important advantage in applications sensitive to high incidence of missing data, such as construction of linkage maps^35^.

Another crucial aspect of MIP performance is their utility as molecular markers. Useful markers may be broadly defined as those reproducibly genotyped and easily interpreted, i.e. are single locus, polymorphic, codominant, and have low incidence of null alleles^36^,^37^. In the SNP literature the fraction of designed markers which can be assayed is termed the conversion rate, while the fraction of markers which are both confirmed as single-locus and polymorphic is referred to as the validation rate. For SNP discovered from transcriptomes both figures are typically lower than those obtained in the current study. For example in a set of fish studies conversion rate ranged between 43 and 92% and validation rate between 12 and 83%; for species without extensive genomic resources these values were in the lower part of the range, unless exon boundaries were identified in transcripts and taken into account during marker design^38^. In species poorly characterized at the genomic level conversion rate will inevitably be variable depending on factors such as intraspecific polymorphism and the genomic rate of duplication. The latter determines the frequency of young paralogs and the extent of copy number variation. In the present study almost all markers were polymorphic in the hybrid family, indicating successful identification of diagnostic positions in transcriptome resequencing data. Although we attempted to filter out paralogs prior to MIP design, the departures from segregation ratios expected under single-locus Mendelian inheritance suggest that ca. 12% of MIP markers may still be paralogs. Additional paralogs were implied by an excess of heterozygotes in *Lvg* populations. Only less than a third of paralogs were common to the two datasets. This may result from high genomic duplication rate and extensive copy number variation within and between newt populations, consistent with the reported ca. 10% differences in genome size between closely related lineages of *L. vulgaris* and *L. montandoni*^34^.

The extent to which MIP markers are transferable between related species is an interesting question. In the present study we assessed performance of MIPs in three populations of *Lvg*, an evolutionary lineage which diverged from *L. vulgaris vulgaris* (*Lvv*) at least 2 mya^39^. Although no genomic information from *Lvg* was used during MIP design, almost all MIPs working in the hybrid family worked also in *Lvg* and 70% were polymorphic. MIPs may be thus more easily transferable between related species than microsatellite loci, especially in species with large genomes^40^,^41^.

The combination of information on performance of MIPs obtained in the present study with previously published data for humans allows assessment of strengths and limitations of the MIP markers in research on non-model organisms. MIPs have a number of advantages:

- They can be reproducibly genotyped from a low amount of input DNA. We used 300–500 ng corresponding to 1–1.6 × 10^4^ template copies and achieved >99% genotype concordance. In humans 50–120 ng of input DNA, i.e. 1.6–4 × 10^4^ copies were used^24^. Thus in species with genomes 1 Gb or smaller, 20–50 ng of genomic DNA should be sufficient. In principle the method should work with even lower amount of input DNA but then the frequency of PCR duplicates increases^42^. If the amount of starting material is limiting, molecular tags uniquely marking reads derived from distinct template molecules may be incorporated into MIP probes to filter out PCR duplicates in downstream bioinformatics analyses^25^,^42^.
- MIP probes are hybridized directly to the extracted genomic DNA, eliminating the need for constructing genomic libraries. Although simplified library construction protocols have been described^43^, preparation of numerous genomic libraries is still laborious, costly and requires microgram DNA quantities, especially if PCR-free protocols are preferred.
- High specificity and extremely high enrichment rate of MIP markers allow genotyping of a relatively small number of targets even in very large and complex genomes. There is much flexibility in this respect: tens, hundreds or thousands of marker can be easily assayed in a single reaction. This is an advantage compared to multiplex PCR assays^44^,^45^,^46^.
- Only standard laboratory equipment is required.
- Workflow is straightforward, the entire procedure can be completed within two working days for hundreds of samples and is amenable to automation using liquid handling systems.
- Thousands of samples can be sequenced simultaneously using dual indexing.
- Design and analysis of MIP markers are relatively simple. Software is available for MIP design^47^ and standard or dedicated^48^ tools may be used for mapping reads to reference and calling polymorphisms.

Molecular Inversion Probes can be either column- or array-synthesized. The former are individually synthesized unmodified oligonucleotides which do not require purification other than standard desalting. Synthesis in a small scale, for example 5 nmol as in our study, is sufficient for virtually unlimited number of samples. Column-synthesized MIPs can be combined into various panels and rebalanced as needed, making the approach extremely versatile. Although the initial cost of probes is considerable if thousands of MIPs are required, it remains constant regardless of the number of samples processed. Handling hundreds or thousands of oligos may be challenging, but is greatly simplified if they are delivered and stored in 96-well plates.

When thousands or tens of thousands of probes are needed they can by synthesized on arrays at an extremely low per probe cost. Oligos are delivered as pool which eliminates the need for handling individual probes. However, this method suffers from serious limitations: i) array-synthesized MIPs are difficult to produce at a scale that would support their use in thousands of samples, ii) the non-uniform synthesis and amplification of array-synthesized MIPs negatively impact the performance of targeted capture, iii) high-quality array-synthesized oligonucleotide libraries are not yet broadly accessible^24^. Array synthesized probes need to be PCR amplified and converted into single stranded probes in a complex, multistep procedure^49^. Recently a simplified procedure utilizing array-synthesized double stranded probes was proposed^42^. Thus it appears that currently both column- and array-synthesized MIP probes are useful, and the choice of either option depends mainly on the target size and the number of samples to process.

MIP markers have also limitations which restrict the range of their applications; the two most important limitations are the target size and cost. If multimegabase regions are targeted, sequence capture techniques would be more efficient due to better uniformity^11^. If probes are column synthesized their cost is substantial, transferring into relatively high per sample cost, especially if few samples are processed. Potentially, the need for prior genomic information required to design markers could be considered a limitation. However, currently sequencing, assembly and detecting polymorphism using transcriptome data are straightforward in any system^50^,^51^.

The cost of column synthesized MIP markers is constant regardless of the number of processed samples. Therefore the per sample and per genotype costs of MIP analysis decrease with the increasing number of samples (Table 2). This effect can be quite dramatic as increasing the number of samples from 100 to 10 000 reduces the per sample cost 20× for 1000 MIPs and 50× for 5000 MIPs. The per genotype cost is $0.017 for 1000 MIPs and 1000 samples and merely $0.002 for 5000 MIPs and 10 000 samples. The calculations in Table 2 exclude sequencing, because many options are available depending on the size of the experiment. Sequencing would however represent a minor fraction of the total cost. For example, assuming that the mean per MIP per sample coverage of 500× is required, sequencing of 1000 MIPs on HiSeq 2500 with would add ca $1.5 per sample or ca $0.0015 per genotype to the total cost.

In conclusion, we demonstrated satisfactory performance of MIP markers designed from transcriptome sequences in a non-model system possessing a very large and complex genome. As few methods for medium-scale targeted resequencing of numerous samples are available for non-model organisms, MIPs fill an important methodological gap. We would thus like to bring the MIP markers to the attention of researchers as a useful extension of the molecular toolkit and an effective solution for large-scale resequencing of tens or hundreds of kb in ecological and evolutionary studies.

## Methods

### Design of Molecular Inversion Probes

**Figure 2:**
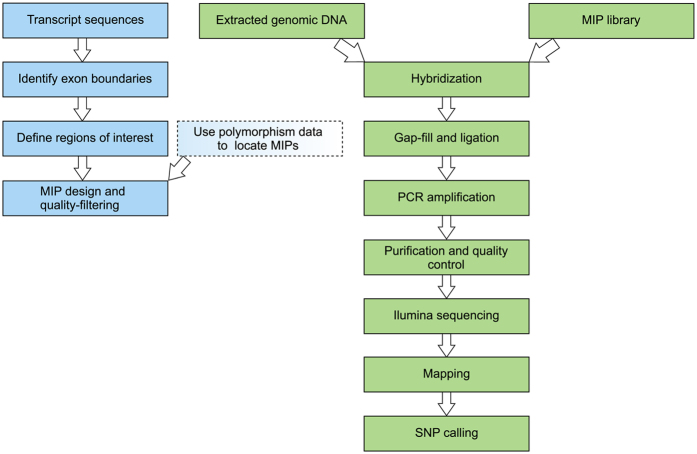
Workflow illustrating the design of Molecular Inversion Probes and laboratory procedures.

**Figure 3:**
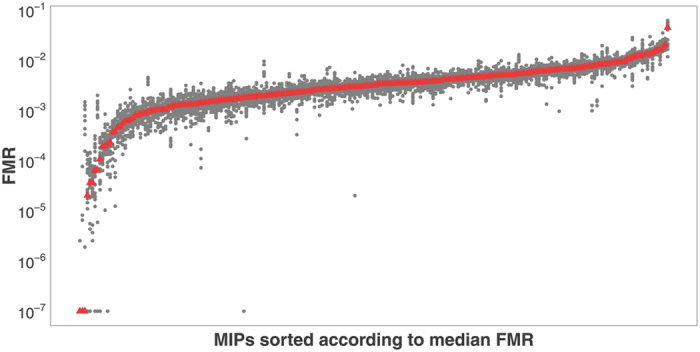
Performance of the MIP markers, data from the experiment 1. Red triangle - median Fraction of Mapped Reads (FMR), gray dots – FMR values for individual newts.

**Figure 4:**
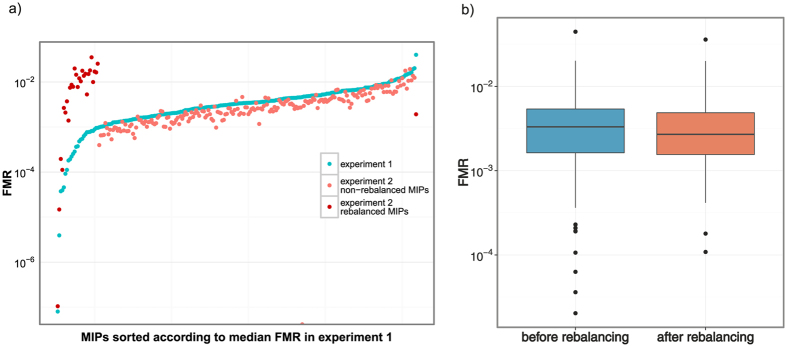
The effect of rebalancing on MIP performance. (**a**) The distribution of the Fraction of Mapped Reads (FMR) before and after rebalancing, (**b**) differences in performance of individual MIPs before and after rebalancing.

Design of Molecular Inversion Probes (MIPs) used in the current study follows that of O’Roak *et al.*^24^ (Fig. 2). Individual MIPs are column synthesized, unmodified, salt-free oligonucleotides 70 bp long. Each MIP contains a common 30 bp linker sequence in the middle, and two fragments complementary to the genomic sequence of interest: extension arm of 16–20 bp at the 3′ end and ligation arm of 20–24 bp at the 5′ end, which together flank a 112 bp target (Fig. 1a). Following hybridization, gap filling and ligation (Fig. 1b), circularized DNA molecules are used as template in PCR with universal primers complementary to the linker sequence (Fig. 1c–e). Sample-specific index sequences and Illumina adaptors are introduced during the PCR step; we used double indexing with 8-bp Nextera indexes. Amplicons are then purified and sequenced using Illumina technology.

In our study two issues had to be taken into account during MIP design. First, because no *Lissotriton* genome is available, MIP markers were designed using transcriptome sequences (available at http://newtbase.eko.uj.edu.pl/). MIP genotyping was however performed from genomic DNA so we had to ensure that individual MIPs are contained within a single exon. To satisfy this requirement we identified exon boundaries in *Lissotriton* transcripts using gene models of *Xenopus tropicalis*, an amphibian species with sequenced and annotated genome. Sequences of *X. tropicalis* exons were downloaded from Ensembl version 79 and used to prepare gene models. *Lissotriton* transcripts were blastn-ed to *X. tropicalis* gene models and exon boundaries were identified in pairwise alignments using scripts available at https://github.com/molecol/targeted-resequencing-with-mips. Only *Lissotriton* contigs mapping unambiguously to single *X. tropicalis* genes were used to minimize the incidence of paralogs. We note that such procedure may introduce bias towards less variable genes and may not be appropriate for some types of studies. Second, MIP markers were developed with the aim of constructing the linkage map of *Lissotriton* newts. Therefore MIPs were selected using available polymorphism data to identify diagnostic markers most informative for the mapping purposes. The linkage map is being constructed using the F2 hybrids between the smooth (*Lissotriton vulgaris vulgaris, Lvv*) and Carpathian (*Lissotriton montandoni, Lm*) newts. Although no transcriptome sequences of parents of the hybrid family (generation P) were available, transcriptomes of two *Lvv* and two *Lm* individuals from the same geographic regions as the P generation newts are available (http://newtbase.eko.uj.edu.pl/). A total of 4,135 contigs representing putative single-copy genes were available for MIP design. This resequencing dataset allowed identification of diagnostic SNPs, i.e. homozygous in all four individuals but with different allele in each species. Although the sample size of two individuals (four gene copies) per species is small, probably these “diagnostic” SNPs indeed have allele frequencies highly differentiated between species and will be useful for mapping because animals of the P generation are likely to be alternative homozygotes.

Regions of interest were defined in BED files and MIP markers were designed using scripts of O’Roak *et al.*^24^ available at: http://krishna.gs.washington.edu/mip_pipeline/; recently, these scripts have been replaced by the MIPgen software^47^, which further optimizes MIP design. To test the impact of the target length on MIP performance, before designing MIPs we randomly deleted codons from 65 contigs; actual targets for MIPs designed in these contigs were longer than the standard 112 bp, varying from 115 to 154 bp and in one case 235 bp. MIPs were ranked according to their predicted performance based on the melting temperature of arms^24^ and only high scoring MIPs encompassing diagnostic SNPs were considered. If multiple MIPs within a gene passed filters, one was randomly selected. In total 248 MIP markers were synthesized (Biosearch Technologies) and tested in the laboratory (Table S1).

### Samples

In total 85 individuals were analyzed. To check the Mendelian inheritance we used a hybrid *Lm* × *Lvv* family, 2 individuals from F1 generation and 21 of their offspring (F2). To test performance of MIPs in a closely related but distinct evolutionary lineage we used samples from three *L. v. graecus* (*Lvg*) populations: Gracen (Albania, 41.16 N, 19.95 E), Milia (Greece, 37.60 N, 22.41 E), and Sagaiika (Greece, 38.10 N, 21.47 E); 18 individuals from each population were analyzed. The remaining 8 individuals were *Lvv* and *Lm* from Poland and additional F2 hybrids from another family. In order to estimate genotyping concordance 16 *Lvg* individuals from the Gracen population were analyzed in replicates.

### Laboratory procedures and sequencing

All experimental protocols were carried out in accordance with the institutional animal ethics permit (number 28/2011) and were approved by the First Local Ethical Committee on Animal Testing at the Jagiellonian University in Krakow. DNA was extracted from tail tips of adults and from whole F2 larvae stored in 96% ethanol using Wizard Genomic DNA Purification Kit (Promega).

DNA was dissolved in 100 ul of TE buffer. Target capture and library construction were performed using the protocol described in Hiatt *et al.*^25^ with modifications during library amplification. Probes were pooled equimolarly and 5′-phosphorylation was performed using 85 ul of the pool, 50 units of T4 Polynucleotide Kinase (NEB) and 10 ul of 10 × T4 DNA ligase buffer in a total volume of 100 ul. Reaction was incubated for 45 min at 37 °C, followed by an inactivation of the kinase at 80 °C for 20 min. Captures were performed using 300–500 ng of genomic DNA, the phosphorylated probe pool at a 1000-fold probe-to-target molar excess (adjusted for the rebalanced pool, see below), and 1 ul of 10 × Ampligase DNA ligase buffer (Epicentre) in a total volume of 10 ul. Hybridization mixture was incubated at 98 °C for 3 min, 85 °C for 30 min, 60 °C for 60 min, and 56 °C for 120 min. Gap filling and ligation reactions contained 10 ul of hybridization mixture, 300 pm of each dNTPs (NEB), 20 nm NAD^+^ (NEB), 7.5 um betaine (Sigma), 1 ul of 10 × Ampligase DNA ligase buffer, 5 units of Ampligase DNA ligase (Epicentre) and 3.2 units of Phusion DNA polymerase (NEB) in a total volume of 20 ul and were carried out at 56 °C for 60 min and 72 °C for 20 min. Reactions were then cooled to 37 °C and 20 units of Exonuclease I (NEB) and 100 units of Exonuclease III (NEB) were added to degrade not circularized probes and genomic DNA. Reactions were incubated at 37 °C for 45 min and at 80 °C for 20 min. For each sample PCR amplification of captured targets was performed using 25 ul of Multiplex PCR Kit (Qiagen), 0.5 uM of each indexed primer, 5 ul of capture reaction and nuclease-free water to 50 ul. The following PCR conditions were used: 95 °C/15 min, 28x (94 °C/30 s, 65 °C/90 s, 72 °C/90 s), 72 °C/10 min. PCR products from multiple samples were pooled equimolarly, run on a 1.5% agarose gel at 6.5 V/cm for 60 min and the band at ca. 270 bp was excised and purified using MinElute Gel Extraction Kit (Qiagen). The purified PCR product was quantified via Qubit and run on a Bioanalyzer (Agilent) to check quality of the library. The library was then diluted to 12 pM and sequenced using custom primers (Table S1) on the MiSeq platform, producing 2 × 150 bp paired-end reads.

### Mapping and genotyping

Reads were mapped to the reference with Bowtie 2^52^ using –end-to-end –sensitive settings. The multisample SNP calling was performed using GenomeAnalysisTK UnifiedGenotyper^53^: PCR error rate was set to 0.005 (–pcr_error_rate 5.0E–3); we excluded all reads with mate unmapped or mapped to a different marker (–read_filter UnmappedRead, –read_filter BadMate); the minimum base quality considered for variant calling was set to 20 (–mbq 20). Genotypes in positions with coverage <16 or genotype quality <30 phred were considered missing. The Fraction of Mapped Reads (FMR) represented by the given MIP within an individual was used as the measure of MIP capture performance.

### Pool rebalancing and statistical analyses

Because MIPs can differ in the capture efficiency, rebalancing was recommended to improve the uniformity of capture as it reduces the required mean coverage and thus minimizes the cost of sequencing^24^. Rebalancing is an adjustment of the concentration of individual probes, in particular increasing the probe-to target ratio for the poorly performing probes. To test the effect of rebalancing MIP capture, amplification and sequencing were performed in two experiments:

1. 24 individuals were analyzed using the MIP pool containing all markers in equal concentration.
2. 77 individuals were analyzed using the rebalanced MIP pool. Based on the results of the experiment 1, 25 MIPs were selected for rebalancing, these were the 24 MIP with the lowest FMR and 1 MIP with the highest FMR; concentrations of the former were increased 100 fold and concentration of the latter was reduced 10 fold.

Sixteen *Lvg* individuals included in both experiments were used to estimate the genotyping error expressed as the non-reference discrepancy rate (NRD)^53^ in GATK module GenotypeConcordance. Mendelian inheritance was tested using the hybrid family. The exact multinomial test in the R package EMT^54^ was applied to test whether genotype counts in progeny followed expectations of the single locus Mendelian inheritance. The effect of rebalancing was assessed with the Levene’s test in the R package car^55^. To identify potential paralogous markers and detect loci with null alleles in the *Lvg* populations genotype frequencies were tested for the agreement with Hardy-Weinberg expectations in Genepop^56^. Markers with an excess of heterozygotes in at least one population were flagged as potential paralogs whereas those with deficit of heterozygotes as loci with null alleles. The number of segregating sites and the nucleotide diversity for three *Lvg* populations were estimated in Arlequin^57^. The remaining statistical tests were performed in R^58^.

## Additional Information

**Accession codes**: MIP and primer sequences: online Supplementary Information. Transcriptome sequences: http://newtbase.eko.uj.edu.pl/. Scripts for identification of exon boundaries: https://github.com/molecol/ targeted-resequencingwith-mips. Scripts for MIP design: http://krishna.gs.washington.edu/mip_pipeline/. Reference sequences, scripts and .vcf files: DRYAD entry doi:10.5061/dryad.q72b7.

**How to cite this article**: Niedzicka, M. *et al.* Molecular Inversion Probes for targeted resequencing in non-model organisms. *Sci. Rep.* **6**, 24051; doi: 10.1038/srep24051 (2016).

## Supplementary Material

Supplementary Information

## Figures and Tables

**Table 1:** Polymorphism in *Lissotriton vulgaris graecus* populations.

	Gracen	Milia	Sagaiika
*S*	224	36	143
π	0.0024	0.0003	0.0021

*S* –number of segregating sites, π –nucleotide diversity.

**Table 2:** Per sample (sample) and per genotype (genotype) costs ($).

N samples	N MIPs					
	100		1000		5000	
	sample	genotype	sample	genotype	sample	genotype
100	16.6	0.166	98.5	0.098	462.5	0.092
1000	8.3	0.083	16.5	0.017	52.9	0.011
10000	7.5	0.075	8.3	0.008	12.0	0.002

These costs include probe synthesis ($9.1 per MIP, does not depend on the number of samples) and reagents for pool phosphorylation, hybridization, gap filling, ligation and PCR amplification ($7.4 per sample, does not depend on the number of MIPs).
