# Supplementary material for: Molecular Inversion Probes for targeted resequencing in non-model organisms
Source: Sci Rep. 2016 Apr 5;6:24051. doi: 10.1038/srep24051 (PMC4820773; doi:10.1038/srep24051)
Supplement: Supplementary Information [file srep24051-s1.pdf]

Supplementary Information for

M. Niedzicka, A. Fijarczyk, K. Dudek, M. Stuglik, W. Babik

Molecular Inversion Probes for targeted resequencing in non-model organisms

This file contains Supplementary Table S1 and Supplementary Figures S1-S2

Supplementary Table S1. Sequences of the Molecular Inversion Probes and primers used in the study.

MIP_ID	extension arm (ea, 5'->3')	ligation arm (la, 5'->3')	full probe sequence (5'->3')	ea length	la length	reference id (available at http://newtbase.eko.uj.edu.pl/)	mip_start on ref.	mip_stop on ref.
057_DGN_10012	TGGAGGCATGTCCCGA	CATCCACATTTTCAATGTCAAGCT	CATCCACATTTTCAATGTCAAGCTCTTCAGCTTCCCGATATCCGACGGTAGTGTTGGAGGCATGTCCCGA	16	24	c102960_g1_i1	758	910
059_DGN_10194	ATACAGATCTATGAGTGT	GTTTCCTCTTGTAGTGTAGCGA	GTTTCCTCTTGTAGTGTAGCGACTTCAGCTTCCCGATATCCGACGGTAGTGTATACAGATCTATGAGTGT	18	22	c128920_g1_i1	2815	2663
060_DGN_10253	GTTGTGTGTATCATGAA	ACTTTCTACTTCAGACAAGTGCA	ACTTTCTACTTCAGACAAGTGCACTTCAGCTTCCCGATATCCGACGGTAGTGTGTTGTGTGTATCATGAA	17	23	c132052_g1_i1	6827	6675
061_DGN_10258	GATCATGGGGAGGTCC	GACTTAGCAGCATTAAGCTCTTCA	GACTTAGCAGCATTAAGCTCTTCACTTCAGCTTCCCGATATCCGACGGTAGTGTGATCATGGGGAGGTCC	16	24	c145157_g1_i1	5035	4882
062_DGN_10274	TGGATGACGTAGATGCCG	CGTTGGGATGGTATCGGCCTTC	CGTTGGGATGGTATCGGCCTTCCTTCAGCTTCCCGATATCCGACGGTAGTGTTGGATGACGTAGATGCCG	18	22	c1357_g1_i1	2350	2190
063_DGN_10276	AAAGTGCACAAACGATA	CCGCAGTACTATAAACGAATCAC	CCGCAGTACTATAAACGAATCACCTTCAGCTTCCCGATATCCGACGGTAGTGTAAAGTGCACAAACGATA	17	23	c89536_g1_i1	624	775
064_DGN_10339	GGAATTTCCAAACTCG	CACTGTACACTCATTTCCTCTTGT	CACTGTACACTCATTTCCTCTTGTCTTCAGCTTCCCGATATCCGACGGTAGTGTGGAATTTCCAAACTCG	16	24	c116585_g1_i1	697	848
065_DGN_10563	CTGCTGCGCATGCTGC	CCTGCTGGCCTCAGCCCTGGCCGT	CCTGCTGGCCTCAGCCCTGGCCGTCTTCAGCTTCCCGATATCCGACGGTAGTGTCTGCTGCGCATGCTGC	16	24	c147128_g1_i1	2369	2211
066_DGN_10700	TGGAATCAACGACAGCCTT	GATGTAGCAAACTCCTTTGGG	GATGTAGCAAACTCCTTTGGGCTTCAGCTTCCCGATATCCGACGGTAGTGTTGGAATCAACGACAGCCTT	19	21	c123361_g1_i1	515	675
067_DGN_10728	GACAGAAGACAAACGTGG	GTCGTGGAGGTCAAAGAGGTGG	GTCGTGGAGGTCAAAGAGGTGGCTTCAGCTTCCCGATATCCGACGGTAGTGTGACAGAAGACAAACGTGG	18	22	c120172_g1_i1	2101	1948
068_DGN_10796	TTCAAGCAGGAATGAG	CACCACGAGGTGGGCGTGGAGGAA	CACCACGAGGTGGGCGTGGAGGAACTTCAGCTTCCCGATATCCGACGGTAGTGTTTCAAGCAGGAATGAG	16	24	c119127_g1_i1	1097	1248
069_DGN_10798	GATGGCAAGCATGACTT	ATGGAGCCCCTTCACCAAAATTT	ATGGAGCCCCTTCACCAAAATTTCTTCAGCTTCCCGATATCCGACGGTAGTGTGATGGCAAGCATGACTT	17	23	c1004_g1_i1	5335	5490
070_DGN_10865	GGATTTACTGCTGCAG	AAGTGCAGCAACATTGAGAACTGA	AAGTGCAGCAACATTGAGAACTGACTTCAGCTTCCCGATATCCGACGGTAGTGTGGATTTACTGCTGCAG	16	24	c112382_g1_i1	941	788
071_DGN_10904	TAGAGGTGTTCATCAC	CCTTGCATGGTAATGCTCCCGCGT	CCTTGCATGGTAATGCTCCCGCGTCTTCAGCTTCCCGATATCCGACGGTAGTGTTAGAGGTGTTCATCAC	16	24	c133767_g1_i1	1675	1831
072_DGN_10960	GACGTTTTAATAAACAA	CACATGATTGAAAGGAAGAAAGG	CACATGATTGAAAGGAAGAAAGGCTTCAGCTTCCCGATATCCGACGGTAGTGTGACGTTTTAATAAACAA	17	23	c120107_g1_i1	1283	1435
073_DGN_10969	TTCTTCTGGGCTCCGAC	GGCCTGCAGAGACAGGCAGCGGG	GGCCTGCAGAGACAGGCAGCGGGCTTCAGCTTCCCGATATCCGACGGTAGTGTTTCTTCTGGGCTCCGAC	17	23	c89163_g1_i1	1082	1233
074_DGN_11012	TGGTTCTGATGGGTTTGTT	CCTACATGTTCGAGATGGATG	CCTACATGTTCGAGATGGATGCTTCAGCTTCCCGATATCCGACGGTAGTGTTGGTTCTGATGGGTTTGTT	19	21	c127465_g1_i1	831	982
075_DGN_11039	CCTGATCTATCAGACCTG	AACAAATGCCTCCGGCAAAAGC	AACAAATGCCTCCGGCAAAAGCCTTCAGCTTCCCGATATCCGACGGTAGTGTCCTGATCTATCAGACCTG	18	22	c94011_g1_i1	2299	2147
076_DGN_11065	CAAGGACTGGTGAAGC	GTTGTTGAACTAAACGATGACAAC	GTTGTTGAACTAAACGATGACAACCTTCAGCTTCCCGATATCCGACGGTAGTGTCAAGGACTGGTGAAGC	16	24	c107593_g1_i1	1726	1889
077_DGN_11096	GACGGTGCCACGTTTT	GGTCAGACTTCGCCAAGCTATCAT	GGTCAGACTTCGCCAAGCTATCATCTTCAGCTTCCCGATATCCGACGGTAGTGTGACGGTGCCACGTTTT	16	24	c136892_g1_i1	2293	2139
078_DGN_11178	ATTCACCAACACGATTA	AAGTGCACAAGTCCTAGGACATC	AAGTGCACAAGTCCTAGGACATCCTTCAGCTTCCCGATATCCGACGGTAGTGTATTCACCAACACGATTA	17	23	c110693_g1_i1	1631	1784
079_DGN_11495	GCATCCTTTTCTCTAGA	ATCTTTCCTGGGCATGGTGGACA	ATCTTTCCTGGGCATGGTGGACACTTCAGCTTCCCGATATCCGACGGTAGTGTGCATCCTTTTCTCTAGA	17	23	c86599_g1_i1	671	519
080_DGN_11535	GATGAGGAAGAACGAGA	GAAAGAAAGGAAGATCAGCAAAC	GAAAGAAAGGAAGATCAGCAAACCTTCAGCTTCCCGATATCCGACGGTAGTGTGATGAGGAAGAACGAGA	17	23	c121276_g1_i1	1842	1999
081_DGN_11555	TTATCAGACTCTTGGAGA	CTGATACATTTGGAGCATGAAC	CTGATACATTTGGAGCATGAACCTTCAGCTTCCCGATATCCGACGGTAGTGTTTATCAGACTCTTGGAGA	18	22	c147657_g1_i1	1814	1660
082_DGN_11575	ATCCTGGTAGGCGATCA	CAGTATCGTATGCATCCTGCGCT	CAGTATCGTATGCATCCTGCGCTCTTCAGCTTCCCGATATCCGACGGTAGTGTATCCTGGTAGGCGATCA	17	23	c129997_g1_i1	2218	2373
084_DGN_11785	GGTGTGTGCCTTGCAGT	CGCGCCGTCCGCTGACTCCTGAC	CGCGCCGTCCGCTGACTCCTGACCTTCAGCTTCCCGATATCCGACGGTAGTGTGGTGTGTGCCTTGCAGT	17	23	c76365_g1_i1	507	659
085_DGN_11820	CACATAAGAGGATTGGC	GTCTTTGTAGAGTGAGTGAGTTG	GTCTTTGTAGAGTGAGTGAGTTGCTTCAGCTTCCCGATATCCGACGGTAGTGTCACATAAGAGGATTGGC	17	23	c130751_g1_i1	3228	3077
086_DGN_11831	ATCCCCTGAAGGACAT	GAAGGCACTACTGCAATTTATATT	GAAGGCACTACTGCAATTTATATTCTTCAGCTTCCCGATATCCGACGGTAGTGTATCCCCTGAAGGACAT	16	24	c131135_g1_i1	522	675
087_DGN_11901	CTGCATTTGAGAATGA	GTGACTGCTTTAGAAGAAGTCCCA	GTGACTGCTTTAGAAGAAGTCCCACTTCAGCTTCCCGATATCCGACGGTAGTGTCTGCATTTGAGAATGA	16	24	c142248_g1_i1	1182	1335
088_DGN_11924	GTCAGATCAATAAAACT	GGAATAGCACTTAATCCGGGAGG	GGAATAGCACTTAATCCGGGAGGCTTCAGCTTCCCGATATCCGACGGTAGTGTGTCAGATCAATAAAACT	17	23	c89994_g1_i1	511	338
089_DGN_11933	TCGCAGACTCCCTTGG	GCCGAAAAGTCCAATGTCAAAATT	GCCGAAAAGTCCAATGTCAAAATTCTTCAGCTTCCCGATATCCGACGGTAGTGTTCGCAGACTCCCTTGG	16	24	c71240_g1_i1	863	1014
090_DGN_11938	GCACAGTTTGAAAGAGAC	CCAAAAAGAAGATGGAGATGGA	CCAAAAAGAAGATGGAGATGGACTTCAGCTTCCCGATATCCGACGGTAGTGTGCACAGTTTGAAAGAGAC	18	22	c121463_g1_i1	3268	3422
091_DGN_12021	AACAGCATGAACGGCTGG	ATGGATGACAGGTCTTGCTTGA	ATGGATGACAGGTCTTGCTTGACTTCAGCTTCCCGATATCCGACGGTAGTGTAACAGCATGAACGGCTGG	18	22	c149098_g1_i1	1413	1262
093_DGN_12071	GACGTCTAGCCCAAAT	GTGCTTGTTGCGATAATACCAAGA	GTGCTTGTTGCGATAATACCAAGACTTCAGCTTCCCGATATCCGACGGTAGTGTGACGTCTAGCCCAAAT	16	24	c88076_g1_i1	333	485
094_DGN_12130	GCTCATCTTTCTGTCC	GTGAACAACATTTCCACACCATTC	GTGAACAACATTTCCACACCATTCCTTCAGCTTCCCGATATCCGACGGTAGTGTGCTCATCTTTCTGTCC	16	24	c76603_g1_i1	1054	903
095_DGN_12257	TCGCCTGTGATTTTGC	CCACTCCCCAGCTCGGTCCACATA	CCACTCCCCAGCTCGGTCCACATACTTCAGCTTCCCGATATCCGACGGTAGTGTTCGCCTGTGATTTTGC	16	24	c110029_g1_i1	519	670
096_DGN_12367	CAGTATAAATGCAAAGTT	CAAGTAATTCATGGCTAATGGG	CAAGTAATTCATGGCTAATGGGCTTCAGCTTCCCGATATCCGACGGTAGTGTCAGTATAAATGCAAAGTT	18	22	c139639_g1_i1	5477	5323
097_DGN_12370	TCACCACTTGAGCTCAGT	GATGTGATGGACTTTGTTGTCA	GATGTGATGGACTTTGTTGTCACTTCAGCTTCCCGATATCCGACGGTAGTGTTCACCACTTGAGCTCAGT	18	22	c100603_g1_i1	777	626
098_DGN_12391	GGCAGGCTTGCTCCCG	GTAGGTTTTCACCTTTGAGATGAT	GTAGGTTTTCACCTTTGAGATGATCTTCAGCTTCCCGATATCCGACGGTAGTGTGGCAGGCTTGCTCCCG	16	24	c120374_g1_i1	5686	5838
0100_DGN_12584	TAGGCTTTGGGGATAGGTTA	ATGCAGTGGAATTGGCAGAG	ATGCAGTGGAATTGGCAGAGCTTCAGCTTCCCGATATCCGACGGTAGTGTTAGGCTTTGGGGATAGGTTA	20	20	c147230_g1_i1	5329	5176
0101_DGN_12615	CTGATTTGAAAAAGAG	CAGGGATTTTTTAAGACCACAAAT	CAGGGATTTTTTAAGACCACAAATCTTCAGCTTCCCGATATCCGACGGTAGTGTCTGATTTGAAAAAGAG	16	24	c90382_g1_i1	1890	1721
0102_DGN_12677	AGATCACATTTGTGAGGT	ACTGTATTCAATCTCCAAAGCC	ACTGTATTCAATCTCCAAAGCCCTTCAGCTTCCCGATATCCGACGGTAGTGTAGATCACATTTGTGAGGT	18	22	c81825_g1_i1	1374	1222
0103_DGN_12808	TACGTTGCCCTTGCTAA	AAGAAGTTCTTCAAAAGCATGAA	AAGAAGTTCTTCAAAAGCATGAACTTCAGCTTCCCGATATCCGACGGTAGTGTTACGTTGCCCTTGCTAA	17	23	c92231_g1_i1	1100	949
0104_DGN_12927	CAAAATCCAGCCTTCC	GGCTTTGGTAGAGAAGCATTAAAA	GGCTTTGGTAGAGAAGCATTAAAACTTCAGCTTCCCGATATCCGACGGTAGTGTCAAAATCCAGCCTTCC	16	24	c110877_g1_i1	1682	1531
0105_DGN_12975	AACAGATACACTGGGA	CAGCTTACTTCTTTTACCAACTCG	CAGCTTACTTCTTTTACCAACTCGCTTCAGCTTCCCGATATCCGACGGTAGTGTAACAGATACACTGGGA	16	24	c152779_g1_i1	1301	1453
0106_DGN_13013	CCCGATTTGGCAGGGC	GCCTGCAGATTTGGAACAACGGCT	GCCTGCAGATTTGGAACAACGGCTCTTCAGCTTCCCGATATCCGACGGTAGTGTCCCGATTTGGCAGGGC	16	24	c117562_g1_i1	2787	2636
0107_DGN_13083	AAGCTCTGGTGCCCGC	ACCAGATGTTTCACATTGAAGGCG	ACCAGATGTTTCACATTGAAGGCGCTTCAGCTTCCCGATATCCGACGGTAGTGTAAGCTCTGGTGCCCGC	16	24	c140576_g1_i1	1255	1419
0108_DGN_13130	CTGGTAGGCCATGTTTCG	GTGGCAGCCACCAAAAATATGT	GTGGCAGCCACCAAAAATATGTCTTCAGCTTCCCGATATCCGACGGTAGTGTCTGGTAGGCCATGTTTCG	18	22	c144030_g1_i1	3816	3661
0109_DGN_13141	GGATTCTGAGGAGCAGG	CAACCTGAGCTACTTTCACACTG	CAACCTGAGCTACTTTCACACTGCTTCAGCTTCCCGATATCCGACGGTAGTGTGGATTCTGAGGAGCAGG	17	23	c152286_g1_i1	1755	1906
0110_DGN_13327	TTGCTTCGCTTCCAATCT	CCTTTGTTGAACGCCTTCTGAA	CCTTTGTTGAACGCCTTCTGAACTTCAGCTTCCCGATATCCGACGGTAGTGTTTGCTTCGCTTCCAATCT	18	22	c81525_g1_i1	1743	1894
0111_DGN_13340	GTCCATGCTTTACTTGGA	CCTAGGGCTTTTAGCACGTCCG	CCTAGGGCTTTTAGCACGTCCGCTTCAGCTTCCCGATATCCGACGGTAGTGTGTCCATGCTTTACTTGGA	18	22	c115367_g1_i1	2836	2685
0112_DGN_13436	GGCGGCGACACTGACA	ACTGCATGAGCTGTATTCAAAACG	ACTGCATGAGCTGTATTCAAAACGCTTCAGCTTCCCGATATCCGACGGTAGTGTGGCGGCGACACTGACA	16	24	c2470_g1_i1	962	1116
0114_DGN_13528	ATATTGTGGATTTCATG	ATTACCTGGATAGCAGAATGAAA	ATTACCTGGATAGCAGAATGAAACTTCAGCTTCCCGATATCCGACGGTAGTGTATATTGTGGATTTCATG	17	23	c149439_g1_i1	618	772
0115_DGN_13641	TTTTTTGTCTTCGACATCA	GCACTGGAGAGACAGGAGAGT	GCACTGGAGAGACAGGAGAGTCTTCAGCTTCCCGATATCCGACGGTAGTGTTTTTTTGTCTTCGACATCA	19	21	c106150_g1_i1	1105	944
0116_DGN_13681	TTCTACGACCTACCTCAG	GCTACCCCATCTTCATCAAGTC	GCTACCCCATCTTCATCAAGTCCTTCAGCTTCCCGATATCCGACGGTAGTGTTTCTACGACCTACCTCAG	18	22	c1842_g1_i1	1578	1751
0117_DGN_13898	ATGGCTGCATCCACCATT	ACTCTTTCGATGCTTCTTCTCT	ACTCTTTCGATGCTTCTTCTCTCTTCAGCTTCCCGATATCCGACGGTAGTGTATGGCTGCATCCACCATT	18	22	c133641_g1_i1	3223	3068
0118_DGN_14064	AGATCAACTCTGATCAGAC	CCCAAACCTAGACGGGGTCGT	CCCAAACCTAGACGGGGTCGTCTTCAGCTTCCCGATATCCGACGGTAGTGTAGATCAACTCTGATCAGAC	19	21	c112000_g1_i1	3830	3989
0119_DGN_14073	GCGTTGTACTTAGTACATT	CACAGTATGGGAAACATCACG	CACAGTATGGGAAACATCACGCTTCAGCTTCCCGATATCCGACGGTAGTGTGCGTTGTACTTAGTACATT	19	21	c120556_g1_i1	4115	3964
0120_DGN_14136	TGTTAATGGGAGTCTAC	CCTCAGAAAGCGCAATTCTCTTC	CCTCAGAAAGCGCAATTCTCTTCCTTCAGCTTCCCGATATCCGACGGTAGTGTTGTTAATGGGAGTCTAC	17	23	c130028_g1_i1	1594	1747
0121_DGN_14244	AAAAGACCTAACAAACA	CAACATGAGCACGTACAGAAATG	CAACATGAGCACGTACAGAAATGCTTCAGCTTCCCGATATCCGACGGTAGTGTAAAAGACCTAACAAACA	17	23	c123522_g1_i1	953	792
0122_DGN_14251	GCTGGAGCTGGTGTGG	GGGCACAGACATGGACACAGGAAT	GGGCACAGACATGGACACAGGAATCTTCAGCTTCCCGATATCCGACGGTAGTGTGCTGGAGCTGGTGTGG	16	24	c146979_g1_i1	2179	2070
0123_DGN_14485	CCATCCTGAATACATCC	CCATTCCATTCCTTTGCTGATGT	CCATTCCATTCCTTTGCTGATGTCTTCAGCTTCCCGATATCCGACGGTAGTGTCCATCCTGAATACATCC	17	23	c248204_g1_i1	2752	2598
0124_DGN_14497	CTCTTCATGAATCTGC	AATCTTGCTTATTATCGTCAATCA	AATCTTGCTTATTATCGTCAATCACTTCAGCTTCCCGATATCCGACGGTAGTGTCTCTTCATGAATCTGC	16	24	c124147_g1_i1	1	123
0125_DGN_14563	ACAATGTCCCTTTATTGGG	GGATACGTGGAAGCAGTGGGT	GGATACGTGGAAGCAGTGGGTCTTCAGCTTCCCGATATCCGACGGTAGTGTACAATGTCCCTTTATTGGG	19	21	c120091_g1_i1	2095	2246
0126_DGN_14586	ATTGGCAACCCCAGAGG	GCTCAGCAAGCAAGGAAGGAAAG	GCTCAGCAAGCAAGGAAGGAAAGCTTCAGCTTCCCGATATCCGACGGTAGTGTATTGGCAACCCCAGAGG	17	23	c121716_g1_i1	2233	2068
0127_DGN_147268	TCAGCCGCATTCTGAT	CATCGCCATTTCTGCTAGCATAAA	CATCGCCATTTCTGCTAGCATAAACTTCAGCTTCCCGATATCCGACGGTAGTGTTCAGCCGCATTCTGAT	16	24	c1387_g1_i1	7790	7638
0128_DGN_14767	GACTGCATGTCAAGTGCT	GCTGCACTTCCACCAGCAGGGC	GCTGCACTTCCACCAGCAGGGCCTTCAGCTTCCCGATATCCGACGGTAGTGTGACTGCATGTCAAGTGCT	18	22	c86553_g1_i1	723	571
0129_DGN_147679	GAGTTCATTTCCAGTA	AGAGGCACACACGTAGTCACGCAT	AGAGGCACACACGTAGTCACGCATCTTCAGCTTCCCGATATCCGACGGTAGTGTGAGTTCATTTCCAGTA	16	24	c106864_g1_i1	2550	2399
0130_DGN_14840	TCCAGTCTCGCCTACC	ACTGTAAATTTATCAATGGCTTCT	ACTGTAAATTTATCAATGGCTTCTCTTCAGCTTCCCGATATCCGACGGTAGTGTTCCAGTCTCGCCTACC	16	24	c148349_g1_i1	354	524
0131_DGN_14847	TTGCTAACGCCTGTATGT	GCGCAGACCTTGTATGCCATTT	GCGCAGACCTTGTATGCCATTTCTTCAGCTTCCCGATATCCGACGGTAGTGTTTGCTAACGCCTGTATGT	18	22	c101767_g1_i1	2698	2849
0132_DGN_15020	TTATCCCACGATACAA	CAATGGATTCTTAATTCCTTTTTG	CAATGGATTCTTAATTCCTTTTTGCTTCAGCTTCCCGATATCCGACGGTAGTGTTTATCCCACGATACAA	16	24	c131559_g1_i1	1298	1449
0133_DGN_15260	TGGTAAAGACTGGGTC	GATGTCTTCAGCTGGTCACAGTAT	GATGTCTTCAGCTGGTCACAGTATCTTCAGCTTCCCGATATCCGACGGTAGTGTTGGTAAAGACTGGGTC	16	24	c142189_g1_i1	1709	1881
0135_DGN_15398	AGAGATGGCTCTTTTGG	GTCCATTCAGGTCCAAGACAACC	GTCCATTCAGGTCCAAGACAACCCTTCAGCTTCCCGATATCCGACGGTAGTGTAGAGATGGCTCTTTTGG	17	23	c123647_g1_i1	1802	1956
0136_DGN_154357	ACATTCTGTTCCATATC	CCAAGTCTTTAACCAGATAGAAT	CCAAGTCTTTAACCAGATAGAATCTTCAGCTTCCCGATATCCGACGGTAGTGTACATTCTGTTCCATATC	17	23	c148821_g1_i1	534	686
0137_DGN_15449	GCACCAGAAGCAGCTGAT	AAGCACATGGCATCAACTGAAC	AAGCACATGGCATCAACTGAACCTTCAGCTTCCCGATATCCGACGGTAGTGTGCACCAGAAGCAGCTGAT	18	22	c247430_g1_i1	2235	2386
0138_DGN_15565	TTTCCGACTTTCAACATT	GCTGTCGGAGCTGGCGGTCCCG	GCTGTCGGAGCTGGCGGTCCCGCTTCAGCTTCCCGATATCCGACGGTAGTGTTTTCCGACTTTCAACATT	18	22	c142015_g1_i1	2812	2659
0139_DGN_15628	AATCCTCAACGTCAAA	GACAATGTTAATGGGTTTCCTATG	GACAATGTTAATGGGTTTCCTATGCTTCAGCTTCCCGATATCCGACGGTAGTGTAATCCTCAACGTCAAA	16	24	c131626_g1_i1	1287	1450
0140_DGN_15696	CTGCACAGAAACTGCGA	GACAGATTGAAAATCGCGAGCGC	GACAGATTGAAAATCGCGAGCGCCTTCAGCTTCCCGATATCCGACGGTAGTGTCTGCACAGAAACTGCGA	17	23	c124093_g1_i1	2001	2160
0141_DGN_15760	TTGCTCTCAGCTTAGGG	ATCGCCAGGTGTCTACCATAGAG	ATCGCCAGGTGTCTACCATAGAGCTTCAGCTTCCCGATATCCGACGGTAGTGTTTGCTCTCAGCTTAGGG	17	23	c137976_g1_i1	1849	1676
0142_DGN_16047	GGGACAGTGAGCACTT	CGAGATGTCATCCATGAGCCACTC	CGAGATGTCATCCATGAGCCACTCCTTCAGCTTCCCGATATCCGACGGTAGTGTGGGACAGTGAGCACTT	16	24	c153996_g1_i1	3195	3044
0143_DGN_16225	GCTTTATTACCACCTGGAG	ACTACCAGAGGCAGCTCATGC	ACTACCAGAGGCAGCTCATGCCTTCAGCTTCCCGATATCCGACGGTAGTGTGCTTTATTACCACCTGGAG	19	21	c137150_g1_i1	675	828
0144_DGN_16319	CACGCCCTCTGCTCCC	CGGTGTGTAACCGATGGCGTGCGC	CGGTGTGTAACCGATGGCGTGCGCCTTCAGCTTCCCGATATCCGACGGTAGTGTCACGCCCTCTGCTCCC	16	24	c106893_g1_i1	864	1017
0145_DGN_16599	CCTGGTGGTATGCCCA	GTAAATGCTTGAGCATGTCCAAGA	GTAAATGCTTGAGCATGTCCAAGACTTCAGCTTCCCGATATCCGACGGTAGTGTCCTGGTGGTATGCCCA	16	24	c123869_g1_i1	2159	2007
0146_DGN_16606	GCGTAGATCTTAAATG	GGATGGAGTCATGATGGTTATCTT	GGATGGAGTCATGATGGTTATCTTCTTCAGCTTCCCGATATCCGACGGTAGTGTGCGTAGATCTTAAATG	16	24	c149066_g1_i1	2161	2333

Supplementary Table S1 continued

MIP_ID	extension arm (ea, 5'->3')	ligation arm (la, 5'->3')	full probe sequence (5'->3')	ea length	la length	reference id (available at http://newtbase.eko.uj.edu.pl/)	mip_start on ref.	mip_stop on ref.
0147_DGN_166235	GCCCAAGGAGCCCGAG	ATAAGCAGCTGAGTCATCTGAACC	ATAAGCAGCTGAGTCATCTGAACCCTTCAGCTTCCCGATATCCGACGGTAGTGTGCCCAAGGAGCCCGAG	16	24	c2238_g1_i1	1452	1620
0148_DGN_16636	TGAACTTTCTTGTATGAA	GTCCTCCCATATAGCCATTCCT	GTCCTCCCATATAGCCATTCCTCTTCAGCTTCCCGATATCCGACGGTAGTGTTGAACTTTCTTGTATGAA	18	22	c147252_g1_i1	4725	4876
0149_DGN_16706	GTTGGAAATTAAGAATACGA	CAAGTGTGACCTTGGAGGTG	CAAGTGTGACCTTGGAGGTGCTTCAGCTTCCCGATATCCGACGGTAGTGTGTTGGAAATTAAGAATACGA	20	20	c149027_g1_i1	1456	1638
0150_DGN_167376	TGCAAACCTCTGGAACAT	AAATCTTCCTTTGCAGAGTCAT	AAATCTTCCTTTGCAGAGTCATCTTCAGCTTCCCGATATCCGACGGTAGTGTTGCAAACCTCTGGAACAT	18	22	c112994_g1_i1	1011	857
0151_DGN_167440	GGGCCAATCAAATGCC	GCCCTGAAGGAGCTGAATGAGCGG	GCCCTGAAGGAGCTGAATGAGCGGCTTCAGCTTCCCGATATCCGACGGTAGTGTGGGCCAATCAAATGCC	16	24	c1111_g1_i1	1125	958
0152_DGN_16786	TCCTCGAAGGGGTTTCC	GCGTTCTCCGCCTGAAAGATGCG	GCGTTCTCCGCCTGAAAGATGCGCTTCAGCTTCCCGATATCCGACGGTAGTGTTCCTCGAAGGGGTTTCC	17	23	c148496_g1_i1	3236	3051
0153_DGN_168704	AACAGGTTTAGGCACTTTA	GGAACCAGCTTCAAGCCTTCT	GGAACCAGCTTCAAGCCTTCTCTTCAGCTTCCCGATATCCGACGGTAGTGTAACAGGTTTAGGCACTTTA	19	21	c137474_g1_i1	496	650
0154_DGN_17005	CGGGATTATGTGTTGCTT	ATGAGAACTCTCCCAGGAATTG	ATGAGAACTCTCCCAGGAATTGCTTCAGCTTCCCGATATCCGACGGTAGTGTCGGGATTATGTGTTGCTT	18	22	c151653_g1_i1	274	434
0155_DGN_1702	GATGGTGGCACAACAACAG	ATCCGAAGAGGAGTCATAGGG	ATCCGAAGAGGAGTCATAGGGCTTCAGCTTCCCGATATCCGACGGTAGTGTGATGGTGGCACAACAACAG	19	21	c110296_g1_i1	619	770
0156_DGN_170323	CCTTCTCCAATATACACCC	GGTGATTCTAGTGGACGGGGA	GGTGATTCTAGTGGACGGGGACTTCAGCTTCCCGATATCCGACGGTAGTGTCCTTCTCCAATATACACCC	19	21	c134379_g1_i1	905	748
0157_DGN_1704	CACGAGGATGATGATTTCA	GATCTTCATTCAGTTGCAGGC	GATCTTCATTCAGTTGCAGGCCTTCAGCTTCCCGATATCCGACGGTAGTGTCACGAGGATGATGATTTCA	19	21	c110873_g1_i1	689	842
0158_DGN_17074	TTTGAAAGCTCTGTATA	GGAGACTATAGTCAGGACCTCCT	GGAGACTATAGTCAGGACCTCCTCTTCAGCTTCCCGATATCCGACGGTAGTGTTTTGAAAGCTCTGTATA	17	23	c151919_g1_i1	3255	3104
0159_DGN_170952	GCAAACTTGTTTTCCAG	GTCACAATTCTATCACTTTTGGG	GTCACAATTCTATCACTTTTGGGCTTCAGCTTCCCGATATCCGACGGTAGTGTGCAAACTTGTTTTCCAG	17	23	c89478_g1_i1	1269	1118
0160_DGN_17202	GTTGGTAGTCTGGGTTGTC	GGGTTCTGGTACAGGTAAGCC	GGGTTCTGGTACAGGTAAGCCCTTCAGCTTCCCGATATCCGACGGTAGTGTGTTGGTAGTCTGGGTTGTC	19	21	c148324_g1_i1	807	652
0161_DGN_172326	GAAACTTGTGACTGCTCA	GTGCTTATTTTGCCACCATTTC	GTGCTTATTTTGCCACCATTTCCTTCAGCTTCCCGATATCCGACGGTAGTGTGAAACTTGTGACTGCTCA	18	22	c148549_g1_i1	1152	985
0162_DGN_173281	ATACATGAAAAAGGACT	CCTCTGCGTAGATAAGCTTCCAA	CCTCTGCGTAGATAAGCTTCCAACTTCAGCTTCCCGATATCCGACGGTAGTGTATACATGAAAAAGGACT	17	23	c125924_g1_i1	2820	2669
0163_DGN_17346	GCTGGTGCAGGAGCCG	CCCTGCACACCCTGATGCACGGCC	CCCTGCACACCCTGATGCACGGCCCTTCAGCTTCCCGATATCCGACGGTAGTGTGCTGGTGCAGGAGCCG	16	24	c153568_g1_i1	471	622
0164_DGN_1743	TGGTAGAACTTTGTCCT	GATCTCTGTTCTCTGTGCGTGAA	GATCTCTGTTCTCTGTGCGTGAACTTCAGCTTCCCGATATCCGACGGTAGTGTTGGTAGAACTTTGTCCT	17	23	c86849_g1_i1	190	341
0165_DGN_174500	CCTCAGCCATTTTGTGAA	GATCAGCCACTTCATGGCCAAG	GATCAGCCACTTCATGGCCAAGCTTCAGCTTCCCGATATCCGACGGTAGTGTCCTCAGCCATTTTGTGAA	18	22	c136975_g1_i1	598	750
0166_DGN_174531	CGCAAGTTAACAGCCGAA	CAGCAATAGAGTCTCTGAAATC	CAGCAATAGAGTCTCTGAAATCCTTCAGCTTCCCGATATCCGACGGTAGTGTCGCAAGTTAACAGCCGAA	18	22	c110073_g1_i1	500	651
0167_DGN_17458	ACAGTGGCAGAAGACAT	GACACACTTATGGAACCATTTTC	GACACACTTATGGAACCATTTTCCTTCAGCTTCCCGATATCCGACGGTAGTGTACAGTGGCAGAAGACAT	17	23	c153820_g1_i1	540	388
0168_DGN_174598	TGTTGGCACTGGAATTTG	CCAGCAGTCTCGAGCTGTTCGC	CCAGCAGTCTCGAGCTGTTCGCCTTCAGCTTCCCGATATCCGACGGTAGTGTTGTTGGCACTGGAATTTG	18	22	c143762_g1_i1	2527	2364
0169_DGN_17520	GTATACGAGATGGCGAGC	GTTTTCACCCACTCTGGTTCTT	GTTTTCACCCACTCTGGTTCTTCTTCAGCTTCCCGATATCCGACGGTAGTGTGTATACGAGATGGCGAGC	18	22	c154335_g1_i1	5842	5690
0170_DGN_176024	TCCATATCTATGAGGTT	GCACTTTTCATCAGAAGTATTGG	GCACTTTTCATCAGAAGTATTGGCTTCAGCTTCCCGATATCCGACGGTAGTGTTCCATATCTATGAGGTT	17	23	c593_g1_i1	903	1054
0171_DGN_176159	CTGGTGATTCGGACGA	CCTCCTGAGGAGGCTGTGACCCTG	CCTCCTGAGGAGGCTGTGACCCTGCTTCAGCTTCCCGATATCCGACGGTAGTGTCTGGTGATTCGGACGA	16	24	c2164_g1_i1	2290	2138
0172_DGN_176305	CGCTCATTTAGTCTCCAAG	ATGCTTGCCGCAGGCTCGAGC	ATGCTTGCCGCAGGCTCGAGCCTTCAGCTTCCCGATATCCGACGGTAGTGTCGCTCATTTAGTCTCCAAG	19	21	c92745_g1_i1	308	465
0173_DGN_176635	GGTCAGGTGCACAAACTCT	GATTGATCGGCGTATGCTGGA	GATTGATCGGCGTATGCTGGACTTCAGCTTCCCGATATCCGACGGTAGTGTGGTCAGGTGCACAAACTCT	19	21	c115777_g1_i1	861	1017
0174_DGN_17768	CTGAACGCCAACTCATT	GTCCTGCTGAAGGAGGCCACCAC	GTCCTGCTGAAGGAGGCCACCACCTTCAGCTTCCCGATATCCGACGGTAGTGTCTGAACGCCAACTCATT	17	23	c114725_g1_i1	1770	1927
0175_DGN_17775	AGCAACCATGGAGATGG	CCTCCGATACCGAAATCCGGTCA	CCTCCGATACCGAAATCCGGTCACTTCAGCTTCCCGATATCCGACGGTAGTGTAGCAACCATGGAGATGG	17	23	c94748_g1_i1	1893	1742
0176_DGN_178307	CCATGATAATTTTCTTG	CAGTGCTTGCCAGCTTTTACGAA	CAGTGCTTGCCAGCTTTTACGAACTTCAGCTTCCCGATATCCGACGGTAGTGTCCATGATAATTTTCTTG	17	23	c152325_g1_i1	4861	4698
0177_DGN_178880	CCTATGGTTTTAACTG	GTCCGATTTTTCAAAGGATACTTT	GTCCGATTTTTCAAAGGATACTTTCTTCAGCTTCCCGATATCCGACGGTAGTGTCCTATGGTTTTAACTG	16	24	c1211_g1_i1	966	1244
0178_DGN_179553	TGAGCTTTTTAGCCCAG	CACTCATCAACGAGTGTTTTATG	CACTCATCAACGAGTGTTTTATGCTTCAGCTTCCCGATATCCGACGGTAGTGTTGAGCTTTTTAGCCCAG	17	23	c142710_g1_i1	744	895
0179_DGN_179771	ACAAAGATCTGATGCT	CCTCAGGGGTCGAAGTCACTGGAC	CCTCAGGGGTCGAAGTCACTGGACCTTCAGCTTCCCGATATCCGACGGTAGTGTACAAAGATCTGATGCT	16	24	c139216_g1_i1	2144	2295
0180_DGN_180053	TTCTCCGTCGCCAACGT	CAGATCTCGGACAAAAAGGCAAA	CAGATCTCGGACAAAAAGGCAAACTTCAGCTTCCCGATATCCGACGGTAGTGTTTCTCCGTCGCCAACGT	17	23	c123846_g1_i1	591	742
0181_DGN_180827	CCCAGATGTTGTCGCA	GACCAGAATGGTACTCTGAACGTG	GACCAGAATGGTACTCTGAACGTGCTTCAGCTTCCCGATATCCGACGGTAGTGTCCCAGATGTTGTCGCA	16	24	c148750_g1_i1	892	1050
0182_DGN_181113	TGTCCTGAATCCATCC	GAGTTGCAAAGAATCACATCTGGA	GAGTTGCAAAGAATCACATCTGGACTTCAGCTTCCCGATATCCGACGGTAGTGTTGTCCTGAATCCATCC	16	24	c151370_g1_i1	3887	3736
0183_DGN_181225	GCCCCACTGTTGGGAA	GTCATCTGGGCCCACTTTTTCAAT	GTCATCTGGGCCCACTTTTTCAATCTTCAGCTTCCCGATATCCGACGGTAGTGTGCCCCACTGTTGGGAA	16	24	c144317_g1_i1	1531	1687
0184_DGN_181327	CTTGCAGGGATGCTTGG	ATGAGGACTCCTGGCAAAGGCGC	ATGAGGACTCCTGGCAAAGGCGCCTTCAGCTTCCCGATATCCGACGGTAGTGTCTTGCAGGGATGCTTGG	17	23	c1827_g1_i1	1100	1252
0185_DGN_181944	TACCTTATGCAGATCATT	ATCTTTACAACCATACGCAAGA	ATCTTTACAACCATACGCAAGACTTCAGCTTCCCGATATCCGACGGTAGTGTTACCTTATGCAGATCATT	18	22	c81395_g1_i1	2757	2603
0186_DGN_182962	TTCAGGTGCTGGATGAT	GGGGCCTTTCCAGCACCAGCTTG	GGGGCCTTTCCAGCACCAGCTTGCTTCAGCTTCCCGATATCCGACGGTAGTGTTTCAGGTGCTGGATGAT	17	23	c133840_g1_i1	3344	3495
0187_DGN_1834	GCAGCTGGAGGACATG	GCAGAGCAGGAGACTCAGTTGGGA	GCAGAGCAGGAGACTCAGTTGGGACTTCAGCTTCCCGATATCCGACGGTAGTGTGCAGCTGGAGGACATG	16	24	c148306_g1_i1	2296	2448
0188_DGN_2247	ATTGATGCGCAGGCAGAA	ACTGGGCGGATGCGGAATGTAA	ACTGGGCGGATGCGGAATGTAACTTCAGCTTCCCGATATCCGACGGTAGTGTATTGATGCGCAGGCAGAA	18	22	c90619_g1_i1	1399	1550
0189_DGN_2404	TGCTTCTCCAGTAAGA	CCATGGGATGAAGTTCTGCTGTCT	CCATGGGATGAAGTTCTGCTGTCTCTTCAGCTTCCCGATATCCGACGGTAGTGTTGCTTCTCCAGTAAGA	16	24	c132762_g1_i1	1	103
0190_DGN_2434	GGAGACAAATCCTTTA	AGACTCCACATCCTGCCGTATCTG	AGACTCCACATCCTGCCGTATCTGCTTCAGCTTCCCGATATCCGACGGTAGTGTGGAGACAAATCCTTTA	16	24	c114826_g1_i1	515	668
0191_DGN_2506	AGGCCCAGCAGATAATGC	GCAGCTACAGAATCGCACCTCC	GCAGCTACAGAATCGCACCTCCCTTCAGCTTCCCGATATCCGACGGTAGTGTAGGCCCAGCAGATAATGC	18	22	c98200_g1_i1	2035	2187
0192_DGN_2611	AAACACTAGTTGCCTT	GTCCAAAGTTCCATTTTTGTGGCA	GTCCAAAGTTCCATTTTTGTGGCACTTCAGCTTCCCGATATCCGACGGTAGTGTAAACACTAGTTGCCTT	16	24	c107425_g1_i1	277	125
0193_DGN_2668	AGTTAGCTGCAACAAGG	CATCCTGAATGGGCTACTTCATT	CATCCTGAATGGGCTACTTCATTCTTCAGCTTCCCGATATCCGACGGTAGTGTAGTTAGCTGCAACAAGG	17	23	c114194_g1_i1	2087	2243
0195_DGN_2710	AATTTGATAATACGCA	GTCTGAGGTTTTTCTGTAATACTC	GTCTGAGGTTTTTCTGTAATACTCCTTCAGCTTCCCGATATCCGACGGTAGTGTAATTTGATAATACGCA	16	24	c124082_g1_i1	2344	2191
0196_DGN_2840	CTTGGTGATCTTCTCTGCC	GTATCTTCTTCTGGACCTGCA	GTATCTTCTTCTGGACCTGCACTTCAGCTTCCCGATATCCGACGGTAGTGTCTTGGTGATCTTCTCTGCC	19	21	c91704_g1_i1	1746	1594
0197_DGN_2856	AGTTGAAAATGAAGACAA	CCAAAAGTTGTCATTGATGGGA	CCAAAAGTTGTCATTGATGGGACTTCAGCTTCCCGATATCCGACGGTAGTGTAGTTGAAAATGAAGACAA	18	22	c144251_g1_i1	961	1112
0198_DGN_2873	TCCGAGACCATGACGGG	GGCTTTGCCTGCCTGCGGGCTTC	GGCTTTGCCTGCCTGCGGGCTTCCTTCAGCTTCCCGATATCCGACGGTAGTGTTCCGAGACCATGACGGG	17	23	c102965_g1_i1	562	714
0199_DGN_2934	TTCTCCCGCGAGCACC	CCGCAACGCCCTCTACCACGTGCA	CCGCAACGCCCTCTACCACGTGCACTTCAGCTTCCCGATATCCGACGGTAGTGTTTCTCCCGCGAGCACC	16	24	c91887_g1_i1	1318	1471
0200_DGN_3034	GTTTCAGCAAAACCAT	CCAGGGGCAGAGATGATCGCCAAA	CCAGGGGCAGAGATGATCGCCAAACTTCAGCTTCCCGATATCCGACGGTAGTGTGTTTCAGCAAAACCAT	16	24	c121045_g1_i1	905	1065
0201_DGN_3060	AAGTCGAAGTATACGGTTT	GCCATCAGGGTCCTCCAAACG	GCCATCAGGGTCCTCCAAACGCTTCAGCTTCCCGATATCCGACGGTAGTGTAAGTCGAAGTATACGGTTT	19	21	c144572_g1_i1	1619	1465
0202_DGN_3121	GCGTAATCCTTCACAATAT	GCTGAGCTCTACGTTGATGGT	GCTGAGCTCTACGTTGATGGTCTTCAGCTTCCCGATATCCGACGGTAGTGTGCGTAATCCTTCACAATAT	19	21	c77798_g1_i1	266	417
0203_DGN_3130	GGCAGGAACCAGACCT	GTTGAAGAGCACGCAGAGCTCGAT	GTTGAAGAGCACGCAGAGCTCGATCTTCAGCTTCCCGATATCCGACGGTAGTGTGGCAGGAACCAGACCT	16	24	c109439_g1_i1	611	763
0204_DGN_3159	CCAATCAGGTCTCATTA	CAATGAGGAGAACTGTATCGAAA	CAATGAGGAGAACTGTATCGAAACTTCAGCTTCCCGATATCCGACGGTAGTGTCCAATCAGGTCTCATTA	17	23	c149145_g1_i1	2393	2241
0205_DGN_3172	TCTCTCCAAAAACAAAGA	ACACACATTTGGGTAGACACCA	ACACACATTTGGGTAGACACCACTTCAGCTTCCCGATATCCGACGGTAGTGTTCTCTCCAAAAACAAAGA	18	22	c121986_g1_i1	1174	1022
0206_DGN_3180	CACTGCTTCCTTGATA	CCAGCTTAGGAAGCTCACACCGAA	CCAGCTTAGGAAGCTCACACCGAACTTCAGCTTCCCGATATCCGACGGTAGTGTCACTGCTTCCTTGATA	16	24	c130654_g1_i1	3257	3102
0207_DGN_3238	TCCAACAGTCATGGGAACG	CGGCTTTGTGGACAGTAGCGA	CGGCTTTGTGGACAGTAGCGACTTCAGCTTCCCGATATCCGACGGTAGTGTTCCAACAGTCATGGGAACG	19	21	c116597_g1_i1	1791	1634
0209_DGN_3285	TGCCAAAATGCTCTGAA	GAAGGAAACCTTCCTGTGTTTGT	GAAGGAAACCTTCCTGTGTTTGTCTTCAGCTTCCCGATATCCGACGGTAGTGTTGCCAAAATGCTCTGAA	17	23	c89898_g1_i1	279	434
0211_DGN_3395	GTACGGATGACGCAGC	CAACGGAAGTGCTCTATGCACCAG	CAACGGAAGTGCTCTATGCACCAGCTTCAGCTTCCCGATATCCGACGGTAGTGTGTACGGATGACGCAGC	16	24	c130723_g1_i1	3707	3858
0212_DGN_3493	TGACTACGACACCCTAACC	CATCACCACTCCACACATGGC	CATCACCACTCCACACATGGCCTTCAGCTTCCCGATATCCGACGGTAGTGTTGACTACGACACCCTAACC	19	21	c121531_g1_i1	3749	3595
0213_DGN_3524	CATCGCGACTCTTGCCC	GGTTTTGCATTTGTTGAATTTGA	GGTTTTGCATTTGTTGAATTTGACTTCAGCTTCCCGATATCCGACGGTAGTGTCATCGCGACTCTTGCCC	17	23	c92694_g1_i1	1145	988
0214_DGN_3552	GACCAGTGGAACTATC	CACACAAGGCTGGGCTGGGAGGTG	CACACAAGGCTGGGCTGGGAGGTGCTTCAGCTTCCCGATATCCGACGGTAGTGTGACCAGTGGAACTATC	16	24	c101424_g1_i1	976	1130
0215_DGN_3616	CATGTTGGCTGCCTGC	CCACCATGTTCCACATCAATGTCC	CCACCATGTTCCACATCAATGTCCCTTCAGCTTCCCGATATCCGACGGTAGTGTCATGTTGGCTGCCTGC	16	24	c144780_g1_i1	668	517
0216_DGN_3694	CTTTCAGACTCTTCCGC	GCTGCTTTCTGAGCAAGGAGCTT	GCTGCTTTCTGAGCAAGGAGCTTCTTCAGCTTCCCGATATCCGACGGTAGTGTCTTTCAGACTCTTCCGC	17	23	c126907_g1_i1	1362	1514
0217_DGN_3731	GTCGTAGTGGTTCGGC	CCACCGTCTTGAAGATTGGCAGCA	CCACCGTCTTGAAGATTGGCAGCACTTCAGCTTCCCGATATCCGACGGTAGTGTGTCGTAGTGGTTCGGC	16	24	c111703_g1_i1	727	880
0218_DGN_3829	CAGTCTCGTTTACTGG	CCCCCAGTTTGTCACGCCATCTTC	CCCCCAGTTTGTCACGCCATCTTCCTTCAGCTTCCCGATATCCGACGGTAGTGTCAGTCTCGTTTACTGG	16	24	c115206_g1_i1	510	359
0219_DGN_3832	TGTGCCATGGGGAAAT	CCTGAGGTCCATCTTCTCCACCAT	CCTGAGGTCCATCTTCTCCACCATCTTCAGCTTCCCGATATCCGACGGTAGTGTTGTGCCATGGGGAAAT	16	24	c132788_g1_i1	517	668
0221_DGN_3969	TACAGTAGTAGCCACAG	GCTTATTGCAACAGTTTGCCCGG	GCTTATTGCAACAGTTTGCCCGGCTTCAGCTTCCCGATATCCGACGGTAGTGTTACAGTAGTAGCCACAG	17	23	c130340_g1_i1	1409	1575
0222_DGN_4007	CTAGGAAGTGCGGGCG	AAGGAGTGGGTCCGCTCCTTCAAG	AAGGAGTGGGTCCGCTCCTTCAAGCTTCAGCTTCCCGATATCCGACGGTAGTGTCTAGGAAGTGCGGGCG	16	24	c115293_g1_i1	885	1036
0223_DGN_4014	CTGACGTGCCTCTGGTT	GCTGCGAATGCGGCTTTCATGCG	GCTGCGAATGCGGCTTTCATGCGCTTCAGCTTCCCGATATCCGACGGTAGTGTCTGACGTGCCTCTGGTT	17	23	c119352_g1_i1	1029	1189
0224_DGN_4039	TTCGATGCAGCATCCT	GGCTCCTGCTCCTCTTCCTTACTC	GGCTCCTGCTCCTCTTCCTTACTCCTTCAGCTTCCCGATATCCGACGGTAGTGTTTCGATGCAGCATCCT	16	24	c127806_g1_i1	3177	3025
0225_DGN_4070	TGCCAGTTTTACAAGCTTG	CAATTTCCGAAAACTCCGTGT	CAATTTCCGAAAACTCCGTGTCTTCAGCTTCCCGATATCCGACGGTAGTGTTGCCAGTTTTACAAGCTTG	19	21	c131046_g1_i1	2026	1857
0226_DGN_4092	TGTTGCAGGCATTTTGTACA	GTCTTTTCGCCAGACTTCGG	GTCTTTTCGCCAGACTTCGGCTTCAGCTTCCCGATATCCGACGGTAGTGTTGTTGCAGGCATTTTGTACA	20	20	c151615_g1_i1	3289	3446
0227_DGN_4109	TTATGATTTTACGCCGATGG	CATGTCTGCCAGTCCACAGC	CATGTCTGCCAGTCCACAGCCTTCAGCTTCCCGATATCCGACGGTAGTGTTTATGATTTTACGCCGATGG	20	20	c118652_g1_i1	1255	1407
0228_DGN_4118	GAGGTCCTCTTCCATC	GCTCTAGTTTTCTTTTCTTCAACA	GCTCTAGTTTTCTTTTCTTCAACACTTCAGCTTCCCGATATCCGACGGTAGTGTGAGGTCCTCTTCCATC	16	24	c945_g1_i1	1207	1358
0229_DGN_4151	TCTGTTGGGCGGGGCC	GAAGATCGATCTCGTGCTGGATGT	GAAGATCGATCTCGTGCTGGATGTCTTCAGCTTCCCGATATCCGACGGTAGTGTTCTGTTGGGCGGGGCC	16	24	c79707_g1_i1	813	662
0230_DGN_4210	TGGAAGACAGTCCGGGC	AGATGCTATCTGACGAAGTCCAA	AGATGCTATCTGACGAAGTCCAACTTCAGCTTCCCGATATCCGACGGTAGTGTTGGAAGACAGTCCGGGC	17	23	c131670_g1_i1	1458	1609
0231_DGN_4211	TGAGAGTTGGCTGAGAGA	CCGTGGTTGCTAAGGAAGGAGG	CCGTGGTTGCTAAGGAAGGAGGCTTCAGCTTCCCGATATCCGACGGTAGTGTTGAGAGTTGGCTGAGAGA	18	22	c145150_g1_i1	332	485
0232_DGN_4212	CGAATTCCTGAAACCTC	CAGATAAGCTGCTCTTCCAGCAA	CAGATAAGCTGCTCTTCCAGCAACTTCAGCTTCCCGATATCCGACGGTAGTGTCGAATTCCTGAAACCTC	17	23	c119643_g1_i1	1291	1105
0233_DGN_4216	GAAATGCAGTAAAAGCTAA	GCCTTACTTTCAGATGTGTCC	GCCTTACTTTCAGATGTGTCCCTTCAGCTTCCCGATATCCGACGGTAGTGTGAAATGCAGTAAAAGCTAA	19	21	c119990_g1_i1	1886	2042
0235_DGN_4245	AGGATATAAATGCATATGAA	CATTGACGTACAGTGCCGGG	CATTGACGTACAGTGCCGGGCTTCAGCTTCCCGATATCCGACGGTAGTGTAGGATATAAATGCATATGAA	20	20	c2393_g1_i1	6792	6637

Supplementary Table S1 continued

MIP_ID	extension arm (ea, 5'->3')	ligation arm (la, 5'->3')	full probe sequence (5'->3')	ea length	la length	reference id (available at http://newtbase.eko.uj.edu.pl/)	mip_start on ref.	mip_stop on ref.
0236_DGN_4416	TGCACTACTGTCGACATT	AATGTGTTGGGAGAAATGCTTG	AATGTGTTGGGAGAAATGCTTGCTTCAGCTTCCCGATATCCGACGGTAGTGTTGCACTACTGTCGACATT	18	22	c2054_g1_i1	8860	8705
0237_DGN_46283	ACATCGCTGTTGTGCA	GGCTTCTCCTCTGAAGATGAGCAT	GGCTTCTCCTCTGAAGATGAGCATCTTCAGCTTCCCGATATCCGACGGTAGTGTACATCGCTGTTGTGCA	16	24	c109325_g1_i1	2404	2253
0239_DGN_4767	CTCCAATGAAGTTGCC	GGAAAGAGGGACTTCTTTTTGTCA	GGAAAGAGGGACTTCTTTTTGTCACTTCAGCTTCCCGATATCCGACGGTAGTGTCTCCAATGAAGTTGCC	16	24	c124581_g1_i1	633	785
0240_DGN_4778	CTTCAGGCGTATGAATT	CCCTCTTGTTGTGCGGACACTAC	CCCTCTTGTTGTGCGGACACTACCTTCAGCTTCCCGATATCCGACGGTAGTGTCTTCAGGCGTATGAATT	17	23	c115230_g1_i1	1320	1169
0241_DGN_4796	TGAAAATGGCAGAAGAGC	CTTCGCCAGCAGCAAGCCTGGA	CTTCGCCAGCAGCAAGCCTGGACTTCAGCTTCCCGATATCCGACGGTAGTGTTGAAAATGGCAGAAGAGC	18	22	c117621_g1_i1	572	723
0242_DGN_4841	GAGTGCTCCATCGTGT	CAGCAAGTACGGGGGGCTGCGCCG	CAGCAAGTACGGGGGGCTGCGCCGCTTCAGCTTCCCGATATCCGACGGTAGTGTGAGTGCTCCATCGTGT	16	24	c139832_g1_i1	1391	1542
0243_DGN_4891	CAGGGTCTTCTCAAAGTC	GTGACCGTCACCGAGCGGCTGA	GTGACCGTCACCGAGCGGCTGACTTCAGCTTCCCGATATCCGACGGTAGTGTCAGGGTCTTCTCAAAGTC	18	22	c132681_g1_i1	1427	1275
0244_DGN_4927	AAACTGGGAGTTGCAG	GTAGAATTGGAGGAACGAGAGTCT	GTAGAATTGGAGGAACGAGAGTCTCTTCAGCTTCCCGATATCCGACGGTAGTGTAAACTGGGAGTTGCAG	16	24	c709_g1_i1	629	781
0245_DGN_5114	CTGCTTGCTGGAGCTG	CTACTACCGAACTATCAAAGGCTT	CTACTACCGAACTATCAAAGGCTTCTTCAGCTTCCCGATATCCGACGGTAGTGTCTGCTTGCTGGAGCTG	16	24	c146268_g1_i1	1581	1734
0246_DGN_5189	TGAAAAACCTTTCAGCTG	ATGCGCAGTGATCACTTGACGA	ATGCGCAGTGATCACTTGACGACTTCAGCTTCCCGATATCCGACGGTAGTGTTGAAAAACCTTTCAGCTG	18	22	c144553_g1_i1	2553	2400
0247_DGN_5192	CGTCACCACTGGTTACA	GGCGCTTCCGCGAACGGTACTCC	GGCGCTTCCGCGAACGGTACTCCCTTCAGCTTCCCGATATCCGACGGTAGTGTCGTCACCACTGGTTACA	17	23	c130485_g1_i1	2109	1957
0248_DGN_5237	CTTCTTTGGCGTTTCGA	GATCTTTGGGGGTTTGGGTTCTT	GATCTTTGGGGGTTTGGGTTCTTCTTCAGCTTCCCGATATCCGACGGTAGTGTCTTCTTTGGCGTTTCGA	17	23	c114595_g1_i1	244	398
0249_DGN_5303	GCCTCAATCAGGTGATCA	GGTATGTGCGTCCTCGCTCTAT	GGTATGTGCGTCCTCGCTCTATCTTCAGCTTCCCGATATCCGACGGTAGTGTGCCTCAATCAGGTGATCA	18	22	c142384_g1_i1	299	147
0250_DGN_5391	TATGTATTTCCGACTTGGA	GACAAGTTCTTGGGTTGCGGA	GACAAGTTCTTGGGTTGCGGACTTCAGCTTCCCGATATCCGACGGTAGTGTTATGTATTTCCGACTTGGA	19	21	c129455_g1_i1	714	868
0251_DGN_5541	TCGTATTCCCACCAGG	GCCGACATCACACTGATTGTTCTG	GCCGACATCACACTGATTGTTCTGCTTCAGCTTCCCGATATCCGACGGTAGTGTTCGTATTCCCACCAGG	16	24	c139748_g1_i1	783	960
0252_DGN_55890	GACCTGGTCCGGCTGC	CACCTTTCACTGGGTTCTGCTGGA	CACCTTTCACTGGGTTCTGCTGGACTTCAGCTTCCCGATATCCGACGGTAGTGTGACCTGGTCCGGCTGC	16	24	c119378_g1_i1	1455	1304
0253_DGN_5645	GCCATCCGCAAAGCCC	GCTTCAGCGAGGCTGCTGCACCAC	GCTTCAGCGAGGCTGCTGCACCACCTTCAGCTTCCCGATATCCGACGGTAGTGTGCCATCCGCAAAGCCC	16	24	c109001_g1_i1	767	918
0254_DGN_5679	CAGCTTGAAGCAGCGCT	GGAGGTGCCACGGCGGCTGTCGC	GGAGGTGCCACGGCGGCTGTCGCCTTCAGCTTCCCGATATCCGACGGTAGTGTCAGCTTGAAGCAGCGCT	17	23	c1017_g1_i1	4485	4636
0255_DGN_5712	ATTCCAATTATCTGGA	AAGAAAAATGGAAGTAAAGTCACA	AAGAAAAATGGAAGTAAAGTCACACTTCAGCTTCCCGATATCCGACGGTAGTGTATTCCAATTATCTGGA	16	24	c141609_g1_i1	1750	1910
0256_DGN_5747	CTTTCCATGGTTTGGCATG	CCAATGTTGCCTATGGTTCCA	CCAATGTTGCCTATGGTTCCACTTCAGCTTCCCGATATCCGACGGTAGTGTCTTTCCATGGTTTGGCATG	19	21	c87136_g1_i1	376	532
0258_DGN_5799	GCCGCAAAAGGAAAATATGA	CGGGGGGATGCTGCCTTACT	CGGGGGGATGCTGCCTTACTCTTCAGCTTCCCGATATCCGACGGTAGTGTGCCGCAAAAGGAAAATATGA	20	20	c148086_g1_i1	2130	2282
0259_DGN_58174	TCGTCTGTCACTTCCCTG	CAGCCTTGGCGCAGAGGTTGGG	CAGCCTTGGCGCAGAGGTTGGGCTTCAGCTTCCCGATATCCGACGGTAGTGTTCGTCTGTCACTTCCCTG	18	22	c73731_g1_i1	841	690
0260_DGN_5829	GCTGAAAGATGAACAGAA	CAACAAGGAATTCCATTATCCG	CAACAAGGAATTCCATTATCCGCTTCAGCTTCCCGATATCCGACGGTAGTGTGCTGAAAGATGAACAGAA	18	22	c145113_g1_i1	2147	2304
0261_DGN_5978	CCAGGAACTAGTGAAAA	GACTGTGGCAGACTTGAAAAAAC	GACTGTGGCAGACTTGAAAAAACCTTCAGCTTCCCGATATCCGACGGTAGTGTCCAGGAACTAGTGAAAA	17	23	c127752_g1_i1	2968	2812
0262_DGN_59978	TGGGAATTCATGGCTTGGC	GCAGTGCGCCAGGATGGCAAT	GCAGTGCGCCAGGATGGCAATCTTCAGCTTCCCGATATCCGACGGTAGTGTTGGGAATTCATGGCTTGGC	19	21	c113322_g1_i1	149	300
0263_DGN_60498	TTCTCAAAATCCTCCTTGT	GATGTAGTATGGATGCGGAGG	GATGTAGTATGGATGCGGAGGCTTCAGCTTCCCGATATCCGACGGTAGTGTTTCTCAAAATCCTCCTTGT	19	21	c143490_g1_i1	5057	4901
0264_DGN_61595	ATCTCCTGTGACTTAA	GGCCATTAAAACAGACTCCTGTCT	GGCCATTAAAACAGACTCCTGTCTCTTCAGCTTCCCGATATCCGACGGTAGTGTATCTCCTGTGACTTAA	16	24	c136375_g1_i1	1374	1525
0265_DGN_6206	TGAATTGTGAGATCTC	GTATCTGTGATGATAACAGTGCCA	GTATCTGTGATGATAACAGTGCCACTTCAGCTTCCCGATATCCGACGGTAGTGTTGAATTGTGAGATCTC	16	24	c136777_g1_i1	577	422
0267_DGN_62259	GGTATCAGGGGGCCTTTCA	GAGGTGAGGTACTAGTGTGGG	GAGGTGAGGTACTAGTGTGGGCTTCAGCTTCCCGATATCCGACGGTAGTGTGGTATCAGGGGGCCTTTCA	19	21	c126492_g1_i1	2039	2195
0268_DGN_6232	TACAGCAAAGTCTAGAGAA	GGGCAACTGGCAGAGCTATTA	GGGCAACTGGCAGAGCTATTACTTCAGCTTCCCGATATCCGACGGTAGTGTTACAGCAAAGTCTAGAGAA	19	21	c138814_g1_i1	1539	1385
0269_DGN_6341	GGGTGGCCCATCATGGT	CCACTGTAGGCTGTGGGATACGC	CCACTGTAGGCTGTGGGATACGCCTTCAGCTTCCCGATATCCGACGGTAGTGTGGGTGGCCCATCATGGT	17	23	c124970_g1_i1	1301	1452
0270_DGN_63510	TGCTCCTGCAGTATGA	GTATCCAGTAGGAGGTAGGTTAAC	GTATCCAGTAGGAGGTAGGTTAACCTTCAGCTTCCCGATATCCGACGGTAGTGTTGCTCCTGCAGTATGA	16	24	c124171_g1_i1	1119	968
0271_DGN_63517	GTTCACATCTGCATTC	GCAGCTGCACAACTTCATCTAAGT	GCAGCTGCACAACTTCATCTAAGTCTTCAGCTTCCCGATATCCGACGGTAGTGTGTTCACATCTGCATTC	16	24	c118259_g1_i1	529	625
0272_DGN_63956	GACTTCTCCCTTGAAAA	GAACATGGATTTTCTGACTCAAC	GAACATGGATTTTCTGACTCAACCTTCAGCTTCCCGATATCCGACGGTAGTGTGACTTCTCCCTTGAAAA	17	23	c118772_g1_i1	302	459
0273_DGN_64226	AGACTGGTAAAACAGTAGC	GGCAGGCTCACGATTGGAAGA	GGCAGGCTCACGATTGGAAGACTTCAGCTTCCCGATATCCGACGGTAGTGTAGACTGGTAAAACAGTAGC	19	21	c127910_g1_i1	1281	1114
0274_DGN_64831	GAAATCAAATGCTCTA	GCACCTTCATATGGTTTAGATGAC	GCACCTTCATATGGTTTAGATGACCTTCAGCTTCCCGATATCCGACGGTAGTGTGAAATCAAATGCTCTA	16	24	c142459_g1_i1	1007	1158
0275_DGN_6516	CAATGAAGAGGCTGAGCA	GATGAGCGAAAAGAGGCACTCT	GATGAGCGAAAAGAGGCACTCTCTTCAGCTTCCCGATATCCGACGGTAGTGTCAATGAAGAGGCTGAGCA	18	22	c151592_g1_i1	3137	2986
0276_DGN_65268	TTTGTCCTTTACCAGGTAA	GACTCAGGTTCTCCAGGTTCT	GACTCAGGTTCTCCAGGTTCTCTTCAGCTTCCCGATATCCGACGGTAGTGTTTTGTCCTTTACCAGGTAA	19	21	c137674_g1_i1	393	510
0277_DGN_6533	AACCTATTGAGGACTCT	CCTCAGGGAATGCCTCAAGGCAT	CCTCAGGGAATGCCTCAAGGCATCTTCAGCTTCCCGATATCCGACGGTAGTGTAACCTATTGAGGACTCT	17	23	c150541_g1_i1	3017	2848
0278_DGN_65619	AATGTGCAGAGCATCACCT	GGAGGAGAAGCGGGGTGGGGG	GGAGGAGAAGCGGGGTGGGGGCTTCAGCTTCCCGATATCCGACGGTAGTGTAATGTGCAGAGCATCACCT	19	21	c131899_g1_i1	1029	1180
0279_DGN_6565	AATGCCTTCTCCAGGAC	GCAGTTTTTTCTACAAGCCATTC	GCAGTTTTTTCTACAAGCCATTCCTTCAGCTTCCCGATATCCGACGGTAGTGTAATGCCTTCTCCAGGAC	17	23	c140448_g1_i1	2648	2490
0280_DGN_6590	GCCTCTGGGGATAAGGGG	GCGGGTCTGCCGCCTCCCCCCG	GCGGGTCTGCCGCCTCCCCCCGCTTCAGCTTCCCGATATCCGACGGTAGTGTGCCTCTGGGGATAAGGGG	18	22	c137744_g1_i1	301	455
0281_DGN_66269	CAACTTCGCCCATTACTT	GTTCGAATCTTTGACCCTGTGA	GTTCGAATCTTTGACCCTGTGACTTCAGCTTCCCGATATCCGACGGTAGTGTCAACTTCGCCCATTACTT	18	22	c145588_g1_i1	505	659
0282_DGN_6664	ACACAGACGACAGAAG	ATGAACACCCTTGCACGTTCTGTA	ATGAACACCCTTGCACGTTCTGTACTTCAGCTTCCCGATATCCGACGGTAGTGTACACAGACGACAGAAG	16	24	c125421_g1_i1	1633	1790
0283_DGN_6707	AGCCCCAGGTAGCCCA	CGGGACGTCTTTGGAGGCTGGGTC	CGGGACGTCTTTGGAGGCTGGGTCCTTCAGCTTCCCGATATCCGACGGTAGTGTAGCCCCAGGTAGCCCA	16	24	c144121_g1_i1	720	880
0284_DGN_672	TCCCACGATCTCTTGGTAG	GAACTGGGCCTTCCACTGCTC	GAACTGGGCCTTCCACTGCTCCTTCAGCTTCCCGATATCCGACGGTAGTGTTCCCACGATCTCTTGGTAG	19	21	c93952_g1_i1	1602	1449
0285_DGN_67736	TTCGCGTATTCCCTGC	CCTCCATCTGGCGGTAACGACTTT	CCTCCATCTGGCGGTAACGACTTTCTTCAGCTTCCCGATATCCGACGGTAGTGTTTCGCGTATTCCCTGC	16	24	c138089_g1_i1	3199	3351
0286_DGN_6794	TCTGATGAAACTAGTTT	GAATCTAATGCTGCTCGCTTCTC	GAATCTAATGCTGCTCGCTTCTCCTTCAGCTTCCCGATATCCGACGGTAGTGTTCTGATGAAACTAGTTT	17	23	c147427_g1_i1	1580	1423
0287_DGN_68266	TATGCCAGGAGGAATT	CATGTTTAGTTATTTCCTGTCATT	CATGTTTAGTTATTTCCTGTCATTCTTCAGCTTCCCGATATCCGACGGTAGTGTTATGCCAGGAGGAATT	16	24	c128093_g1_i1	676	860
0288_DGN_68680	TCTTCAGTTTCATCGT	GCCTTCTGGAATTCTGGGTGATCG	GCCTTCTGGAATTCTGGGTGATCGCTTCAGCTTCCCGATATCCGACGGTAGTGTTCTTCAGTTTCATCGT	16	24	c130037_g1_i1	353	506
0289_DGN_68716	TCCGGAAGTATGGTAGG	GAGGCCAACATTGGATTCAGTGA	GAGGCCAACATTGGATTCAGTGACTTCAGCTTCCCGATATCCGACGGTAGTGTTCCGGAAGTATGGTAGG	17	23	c120339_g1_i1	1489	1336
0290_DGN_69682	GATAGAGTTGTGGTCGA	ATGATTTCTACACCATCTCCGAA	ATGATTTCTACACCATCTCCGAACTTCAGCTTCCCGATATCCGACGGTAGTGTGATAGAGTTGTGGTCGA	17	23	c53956_g1_i1	359	208
0291_DGN_70436	TTGCCTTTTCCACCAGC	ACGCTAGTCAACCCCTCAATGCT	ACGCTAGTCAACCCCTCAATGCTCTTCAGCTTCCCGATATCCGACGGTAGTGTTTGCCTTTTCCACCAGC	17	23	c127768_g1_i1	1765	1920
0292_DGN_7045	AGGTAGTTAAATACTGTG	CGGAGGTGCAGAACATGCCAGC	CGGAGGTGCAGAACATGCCAGCCTTCAGCTTCCCGATATCCGACGGTAGTGTAGGTAGTTAAATACTGTG	18	22	c146704_g1_i1	1716	1630
0293_DGN_7054	CCTTCAGGAATTCCTGTTC	GCCCATGGGTAATTGACCAGA	GCCCATGGGTAATTGACCAGACTTCAGCTTCCCGATATCCGACGGTAGTGTCCTTCAGGAATTCCTGTTC	19	21	c2890_g1_i1	5603	5452
0294_DGN_7078	GAGTCAGAGACGCTACG	GATGCTGATTGGCTAGGCTGCTC	GATGCTGATTGGCTAGGCTGCTCCTTCAGCTTCCCGATATCCGACGGTAGTGTGAGTCAGAGACGCTACG	17	23	c149269_g1_i1	13366	13518
0295_DGN_7171	CTCACGCAGCGCCTTC	GAAGAGCACAGTGGAGCTTTTAAC	GAAGAGCACAGTGGAGCTTTTAACCTTCAGCTTCCCGATATCCGACGGTAGTGTCTCACGCAGCGCCTTC	16	24	c138319_g1_i1	705	554
0296_DGN_71802	AGAACGCGCTTTCCAAAA	GCAAAAGGTCTGTTTGAACAAA	GCAAAAGGTCTGTTTGAACAAACTTCAGCTTCCCGATATCCGACGGTAGTGTAGAACGCGCTTTCCAAAA	18	22	c141309_g1_i1	2103	2258
0297_DGN_7197	AACACCATAAGCCGCT	ATCCTCCTCCAGGAGCACAGCCTC	ATCCTCCTCCAGGAGCACAGCCTCCTTCAGCTTCCCGATATCCGACGGTAGTGTAACACCATAAGCCGCT	16	24	c151655_g1_i1	2039	2190
0298_DGN_72205	CATGCACAGCCACCCA	CGCTTAGGCCGGAAAGTGCGATCT	CGCTTAGGCCGGAAAGTGCGATCTCTTCAGCTTCCCGATATCCGACGGTAGTGTCATGCACAGCCACCCA	16	24	c132735_g1_i1	3107	2949
0299_DGN_72649	TGTGACAGGCATGGGCAC	GCTACCTCATCCAGCCAACGGT	GCTACCTCATCCAGCCAACGGTCTTCAGCTTCCCGATATCCGACGGTAGTGTTGTGACAGGCATGGGCAC	18	22	c125155_g1_i1	1687	1854
0301_DGN_73139	TTTCTTCTCCTCCATG	CATTTTCACAACCTGGCTGGGTAG	CATTTTCACAACCTGGCTGGGTAGCTTCAGCTTCCCGATATCCGACGGTAGTGTTTTCTTCTCCTCCATG	16	24	c149702_g1_i1	4189	4038
0302_DGN_73299	AGTAGGTTTTGACAACGTCA	CAAATGGCTTTCCAGCCAGG	CAAATGGCTTTCCAGCCAGGCTTCAGCTTCCCGATATCCGACGGTAGTGTAGTAGGTTTTGACAACGTCA	20	20	c145460_g1_i1	964	1119
0303_DGN_74221	TTCCAGCAGCTCCTGT	ACAGTTGATAGTTCCAGCTAATGG	ACAGTTGATAGTTCCAGCTAATGGCTTCAGCTTCCCGATATCCGACGGTAGTGTTTCCAGCAGCTCCTGT	16	24	c153897_g1_i1	1561	1716
0304_DGN_75071	TGGATTCCAAACACTT	CCACAATTGAACATACACTTTGCA	CCACAATTGAACATACACTTTGCACTTCAGCTTCCCGATATCCGACGGTAGTGTTGGATTCCAAACACTT	16	24	c143810_g1_i1	2645	2490
0305_DGN_75073	CACCCCAGCCTCTCTCA	CCCATGAATGGAAGCCAGCCCTT	CCCATGAATGGAAGCCAGCCCTTCTTCAGCTTCCCGATATCCGACGGTAGTGTCACCCCAGCCTCTCTCA	17	23	c1556_g1_i1	3969	3818
0306_DGN_75311	GAAATCCCTACTCTCCCTT	ACTCGGACACTGGATTTTCTG	ACTCGGACACTGGATTTTCTGCTTCAGCTTCCCGATATCCGACGGTAGTGTGAAATCCCTACTCTCCCTT	19	21	c148890_g1_i1	1995	2147
0307_DGN_76507	CGGTGCCGTGGGAGGA	CACCACTTCGGGTATGGCCTGGCC	CACCACTTCGGGTATGGCCTGGCCCTTCAGCTTCCCGATATCCGACGGTAGTGTCGGTGCCGTGGGAGGA	16	24	c142945_g1_i1	575	730
0308_DGN_77506	CAGTATTTCAGAATTTTC	GAGATGGAAATATTGTGAAGGC	GAGATGGAAATATTGTGAAGGCCTTCAGCTTCCCGATATCCGACGGTAGTGTCAGTATTTCAGAATTTTC	18	22	c152145_g1_i1	1039	1190
0309_DGN_77541	ATGGTGTTCAGGAAGT	GAGGTGCTCCAGCATGATGGAGGC	GAGGTGCTCCAGCATGATGGAGGCCTTCAGCTTCCCGATATCCGACGGTAGTGTATGGTGTTCAGGAAGT	16	24	c2298_g1_i1	911	1062
0310_DGN_77571	ATAGATTTCAAGGCCT	GGTAGATGCGGATAACCTGCTGAA	GGTAGATGCGGATAACCTGCTGAACTTCAGCTTCCCGATATCCGACGGTAGTGTATAGATTTCAAGGCCT	16	24	c146798_g1_i1	3159	3311
0311_DGN_77620	TTTTCTTTTTCCTCCTCA	ATGGTGCAGGGGTGCTAGTGGT	ATGGTGCAGGGGTGCTAGTGGTCTTCAGCTTCCCGATATCCGACGGTAGTGTTTTTCTTTTTCCTCCTCA	18	22	c141619_g1_i1	553	712
0312_DGN_77747	TTCAGGAAGTTTACGAATT	GTAGAGAAGAAAGCCGACCAG	GTAGAGAAGAAAGCCGACCAGCTTCAGCTTCCCGATATCCGACGGTAGTGTTTCAGGAAGTTTACGAATT	19	21	c152047_g1_i1	1100	1293
0313_DGN_77795	ATTTGATATCCGTTATTA	ATGTCATGTCATAGCCATCAGG	ATGTCATGTCATAGCCATCAGGCTTCAGCTTCCCGATATCCGACGGTAGTGTATTTGATATCCGTTATTA	18	22	c150073_g1_i1	1666	1515
0314_DGN_77801	TACTCTCACCGCTATGCAC	CGCCAGCGACCTCTATGACTC	CGCCAGCGACCTCTATGACTCCTTCAGCTTCCCGATATCCGACGGTAGTGTTACTCTCACCGCTATGCAC	19	21	c136849_g1_i1	886	735
0315_DGN_77934	GACTTGGCTTGGAAACA	ACCATGGTGGGACTCATTTTAGG	ACCATGGTGGGACTCATTTTAGGCTTCAGCTTCCCGATATCCGACGGTAGTGTGACTTGGCTTGGAAACA	17	23	c753_g1_i1	807	958
0316_DGN_78272	GGCTACAGCCATGGAA	GCTTCGAAGCTAGCTCCTGGGTGT	GCTTCGAAGCTAGCTCCTGGGTGTCTTCAGCTTCCCGATATCCGACGGTAGTGTGGCTACAGCCATGGAA	16	24	c153632_g1_i1	5770	5617
0317_DGN_78342	CAAATACAAAGGTATTC	GCATTGAGATAATTAAAGATCGC	GCATTGAGATAATTAAAGATCGCCTTCAGCTTCCCGATATCCGACGGTAGTGTCAAATACAAAGGTATTC	17	23	c154819_g1_i1	11491	11334
0318_DGN_85973	AGGTCACGTTTCTTGTC	AGAAGTGGTGTCATGCTGTAGCC	AGAAGTGGTGTCATGCTGTAGCCCTTCAGCTTCCCGATATCCGACGGTAGTGTAGGTCACGTTTCTTGTC	17	23	c99132_g1_i1	1464	1616
0319_DGN_9497	CTTTGTTCCACAAACCACTT	GTGGTAGCCGGAATCACTCT	GTGGTAGCCGGAATCACTCTCTTCAGCTTCCCGATATCCGACGGTAGTGTCTTTGTTCCACAAACCACTT	20	20	c102837_g1_i1	1996	1845

Primers used for amplification and sequencing

**Type**	**Name**	**Sequence**	**(5'->3')**
PCR	amplification	MIP_FOR	AATGATACGGCGACCACCGAGATCTACACNNNNNNNNCATACGAGATCCGTAATCGGGAAGCTGAAG
PCR	amplification	MIP_REV	CAAGCAGAAGACGGCATACGAGATNNNNNNNNACACGCACGATCCGACGGTAGTGT
Sequencing	MIP_SEQ_FOR	CATACGAGATCCGTAATCGGGAAGCTGAAG	
Sequencing	MIP_SEQ_REV	ACACGCACGATCCGACGGTAGTGT	
Sequencing	MIP_SEQ_INDX	ACACTACCGTCGGATCGTGCGTGT	
NNNNNNNN	Nextera Index Sequence		

Supplementary Figures

Fig. S1. The image of an agarose gel showing amplification products of non-rebalanced and rebalanced pools; note increased amount of nonspecific amplification products in rebalanced pools.

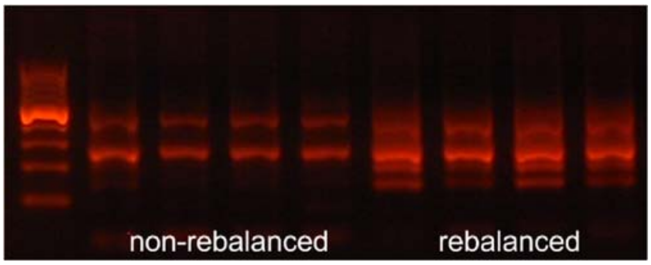

Fig. S2. The Bioanalyzer traces showing non-rebalanced and rebalanced pools after gel purification targeting the 270 bp fragment.

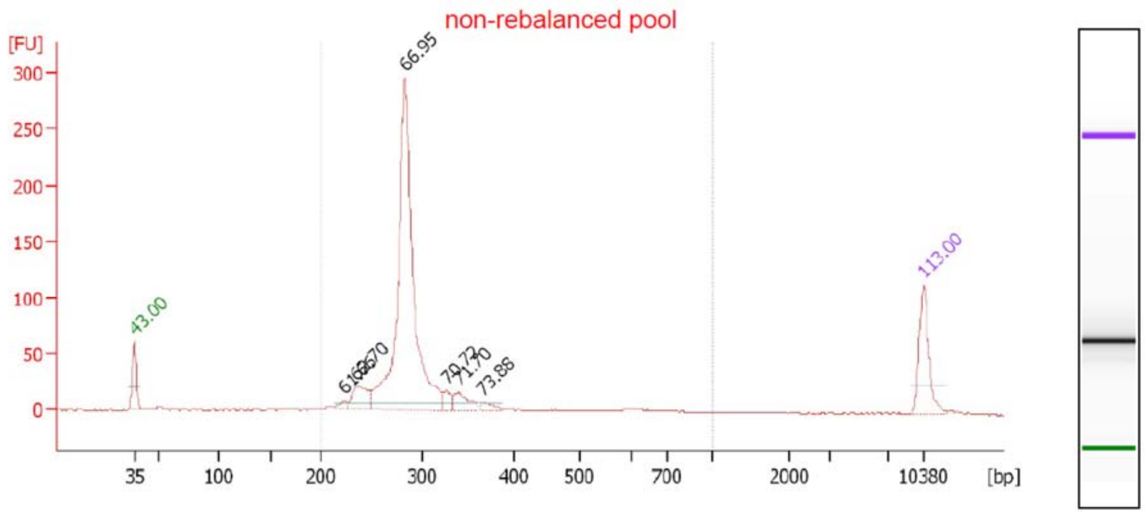

Overall Results for sample 2 : __mip__

Number of peaks found:	6	Corr. Area 1:	909.4
Noise:	0.3		

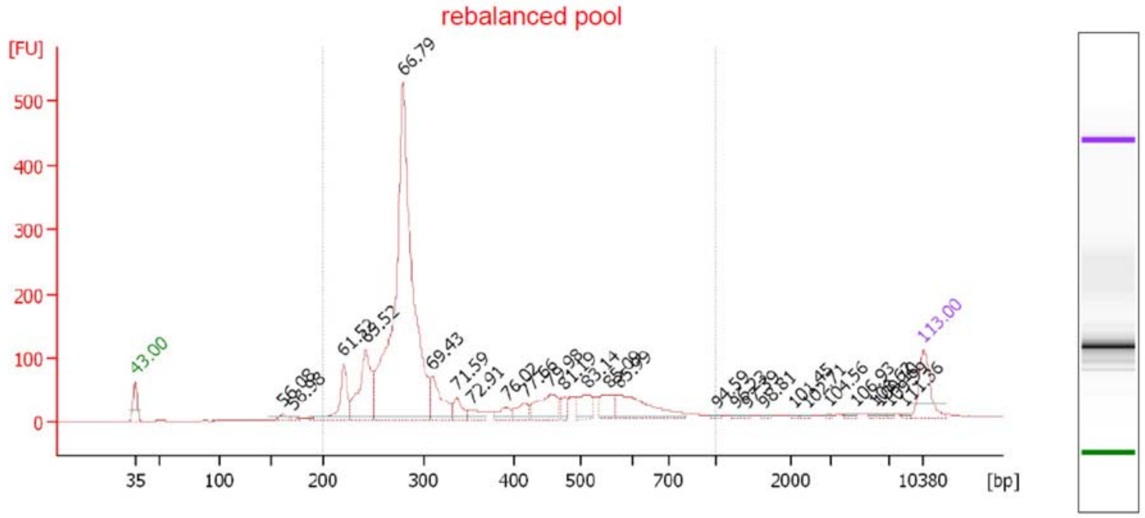

Overall Results for sample 1 : __MIP__

Number of peaks found:	27	Corr. Area 1:	2,627.8
Noise:	0.4		
